# Bone-seeking nanomaterials for rebalancing bone remodeling in osteoporosis

**DOI:** 10.1016/j.ijpx.2026.100578

**Published:** 2026-05-29

**Authors:** Fei Wang, Feng Liang, Xiaonan Zhou, Yuanyuan Ding, Mingzhe Wu, Nan Li, Fei Wang, Wei Yan

**Affiliations:** a Department of Orthopedics, Shengjing Hospital of China Medical University, Shenyang, Liaoning Province, China; b Department of Pain, Shengjing Hospital of China Medical University, Shenyang, Liaoning Province, China; c Department of Gynecology, The First Hospital of China Medical University, Shenyang, Liaoning Province, China; d Department of Pediatric, The Fourth Affiliated Hospital of China Medical University, Shenyang, Liaoning Province, China; e Department of Otolaryngology, The First Hospital of China Medical University, Shenyang, Liaoning Province, China; f Department of Pediatric Orthopedics, Shengjing Hospital of China Medical University, Shenyang, Liaoning Province, China

**Keywords:** Osteoporosis, Bone-seeking nanomaterials, Bone-targeted drug delivery, Osteoclast–osteoblast coupling, Bone regeneration

## Abstract

Osteoporosis is a prevalent skeletal disorder characterized by reduced bone mass and microarchitectural deterioration, primarily resulting from an imbalance between osteoclast-mediated bone resorption and osteoblast-mediated bone formation. Although current anti-osteoporosis drugs can slow bone loss or stimulate bone formation, their clinical effectiveness is often limited by poor bone specificity, systemic side effects, and insufficient restoration of physiological bone remodeling. Recently, bone-seeking nanomaterials have emerged as a promising strategy to enhance therapeutic precision in osteoporosis. By integrating bone-targeting ligands with engineered nanocarriers, these systems enable preferential accumulation in mineralized tissues and improve the local delivery of therapeutic agents. In addition to conventional anti-resorptive and anabolic drugs, nanoplatforms have been developed to deliver nucleic acids, growth factors, and immunomodulatory molecules that regulate the complex cellular networks governing bone remodeling. This review summarizes recent advances in bone-targeted nanomaterial design, discusses their roles in modulating osteoclasts, osteoblasts, osteocytes, and osteoimmune interactions, and highlights key challenges and future opportunities for the clinical translation of nanomedicine in osteoporosis therapy. Distinct from prior reviews that often emphasize nanocarrier composition or individual payload classes, this review is organized around the concept of rebalancing bone remodeling and integrates osteoclast, osteoblast, osteocyte, and osteoimmune regulation with emerging translational considerations.

## Highlights

• This review details bone-targeted ligand design for precise drug delivery to osteoporosis sites.
• It evaluates diverse nanoplatforms (lipid, polymer, inorganic) for multi-agent therapy.
• The work highlights nanomaterial-mediated multi-cellular regulation of bone remodeling.
• It covers advanced strategies like gene/RNA nanomedicine and stimuli-responsive systems.

## Graphical abstract

Bone-targeted nanotherapies restore bone remodeling balance in osteoporosis. Osteoporosis is characterized by excessive osteoclast activity driven by the RANKL/NF-κB signaling together with impaired osteoblast function associated with reduced BMP-2, Wnt/β-catenin, and Runx2 activity, resulting in an imbalance between bone resorption and bone formation. Bone-targeted nanotherapeutic platforms, including lipid and inorganic nanoparticles delivering siRNA, BMP-2, or anti-RANKL agents, suppress osteoclast-mediated bone resorption, enhance osteoblast-mediated bone formation, and ultimately promote bone regeneration and restoration of skeletal homeostasis.

**Figure:**
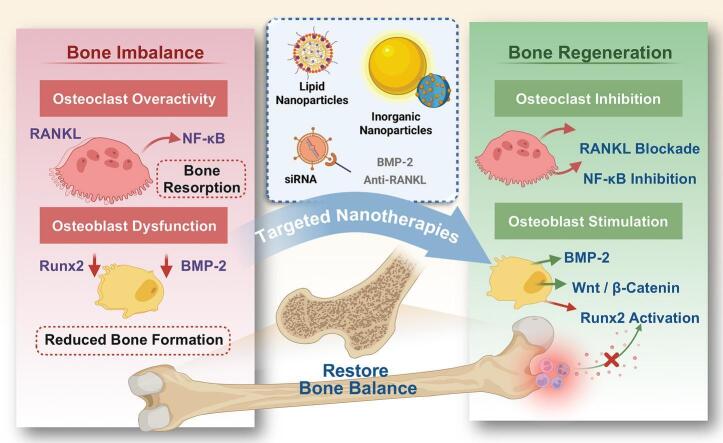

## 1 Introduction

Osteoporosis has become one of the most common chronic bone diseases in the world. The disease affects hundreds of millions of people and is one of the main causes of disability among the elderly (Muñoz et al., 2020; Subarajan et al., 2024). Many countries are facing a rapid increase in the number of patients with osteoporosis fractures, especially with the increasing life expectancy. Epidemiological studies show that one-third of women and one-fifth of men experience osteoporotic fractures in their lifetime (Anam and Insogna, 2021; Tella and Gallagher, 2014). These fractures often occur in the hips, spine and wrists. Hip fractures are particularly destructive and often lead to increased long-term disability and mortality. The social and economic impact of osteoporosis is huge. The health care system spends billions of dollars a year on fracture treatment, rehabilitation and long-term care (Liao et al., 2023, Shi et al., 2025). Patients often suffer from chronic pain and mobility difficulties. Many patients have lost the ability to live independently after a serious fracture (Black and Rosen, 2016; Cortet et al., 2024). These consequences have put a heavy burden on families and caregivers. This is a global challenge.

The researchers also observed significant changes in the osteoporotic fractures. Population aging is still the most important driving factor. However, lifestyle factors can also exacerbate the progression of the disease. Reduced physical activity, nutritional imbalance, and hormonal changes will accelerate bone loss (Gao and Zhao, 2023; Cosman, 2009). In addition, metabolic diseases and chronic inflammation can further destroy bone homeosis. These factors work together to aggravate bone fragility. Since fractures are largely preventable, people's scientific interest in osteoporosis continues to grow. Early intervention can significantly reduce the risk of bone degeneration (Fleseriu, 2018; Capozzi et al., 2014). However, effective prevention requires an in-depth understanding of the kinetics of bone remodeling and the molecular network that regulates bone homeostasis. Therefore, many researchers are committed to the biological process of maintaining bone integrity. Understanding these mechanisms lays the foundation for developing more effective treatment strategies.

Bone tissue is constantly renewed all life. This process is called bone remodeling. Two main cell groups control this dynamic cycle. Osteoclasts remove old bones through resorption, while osteoblasts produce new bone matrix and promote mineralization. The synergistic activities of these two cells maintain the strength and structural stability of the bone (Li et al., 2020; Lerner, 2006). Healthy bones maintain a balance between bone formation and bone resorption. When this balance is broken, osteoporosis will occur. Too high osteoclast activity will accelerate bone degradation (Liao et al., 2024, Liao et al., 2025). At the same time, impaired osteoblast function will limit the formation of new bone tissue. The result is a gradual decrease in bone mass and deterioration of bone microstructure. The bones become porous and fragile. The precise coordination between bone cells is governed by interconnected signaling networks that collectively regulate osteoclast differentiation, osteoblast activity, and osteocyte-mediated communication. The major molecular pathways relevant to osteoporosis, including RANKL/RANK/OPG, Wnt/β-catenin, BMP, and inflammatory signaling (Tao et al., 2021; Jiang et al., 2024; Rong et al., 2022; Oh et al., 2023; Cheng et al., 2022; Huang et al., 2022).

Bone cells are another important participant in the process of bone remodeling. Bone cells are mature bone cells embedded in the mineralized matrix. These cells are like mechanical receptors, which can sense mechanical stress and convert physical signals into biochemical reactions. Bone cells also regulate the secretion of signal molecules such as osteoprotein, which can inhibit the activity of osteoblasts (Cheng et al., 2022; Huang et al., 2022). Therefore, bone cell dysfunction will destroy the communication network that coordinates bone remodeling. Current research increasingly emphasizes the complexity of bone homeostasis. Bone remodeling is not only a cellular process, but also involves the interaction between the immune system, vascular networks and metabolic signals. The concept of this integration is called bone immunology. These interactions highlight the need to develop therapies that can act on multiple regulatory pathways at the same time.

Current pharmacological management of osteoporosis primarily targets either bone resorption or bone formation. Anti-resorptive agents, such as bisphosphonates, denosumab, and selective estrogen receptor modulators, are widely used in clinical practice. They work by effectively reducing osteoclast activity, thereby slowing bone loss (Huang et al., 2025). However, these therapies cannot fully restore physiological bone remodeling. A primary limitation is that prolonged suppression of bone turnover may adversely affect bone material properties. Anabolic therapy represents another cornerstone of treatment (Ramchand and Leder, 2024; Wu et al., 2021). Agents like teriparatide and romosozumab stimulate osteoblast activity, promoting bone formation, increasing bone mineral density, and reducing fracture risk. Despite their efficacy, challenges remain. Many anabolic drugs require repeated subcutaneous injection. Due to safety considerations, their use is subject to a limited treatment duration. Furthermore, the anabolic effect gradually diminishes after therapy cessation.

Poor tissue specificity is still the main limitation of existing drugs. Most traditional osteoporosis drugs will be distributed throughout the body after systemic administration. This non-specific distribution usually reduces the therapeutic effect (Li et al., 2025, Liao et al., 2026, Liao et al., 2026). Only a very small part of the drug can reach the skeletal tissue. Non-targeted accumulation may lead to adverse side effects. Some anti-resorptive drugs have been reported to cause gastrointestinal irritation and jawbone necrosis (Rachner et al., 2011; Cosman, 2014). Another major challenge is to restore the balance of bone remodeling. The current therapy is usually aimed at a single pathway. However, osteoporosis involves multiple regulatory networks. Effective treatment requires the simultaneous regulation of osteoclasts, osteoblasts, and osteocytes. Traditional drugs can rarely achieve this kind of integrated regulation. Therefore, the normal physiological function of bones still cannot be fully restored. Therefore, researchers realize that more accurate treatment strategies are needed. Future treatments must improve bone targeting and realize the controlled release of therapeutic molecules. Achieving these goals is expected to significantly improve the treatment effect of osteoporosis.

The progress of nanomedicine has opened up new opportunities for the treatment of bone diseases. Nanotechnology enables scientists to design drug carriers on a nanoscale (Liao et al., 2026, Liao et al., 2026, Pan et al., 2025). These carriers can protect therapeutic molecules and improve their pharmacokinetic behavior. Nanoparticles can also be modified by targeted ligands. These characteristics enhance the delivery of drugs to specific tissues (Qiao et al., 2022; Cui et al., 2022a). Many researchers are exploring the application of nanomaterials in bone-related diseases. Nanoparticles can encapsulate small molecules, proteins, or nucleic acids. These carriers can protect sensitive therapeutic drugs from degradation during circulation. The controllable release mechanism can regulate the exposure of drugs over time. These characteristics improve the stability and efficacy of treatment.

Nanotechnology can also develop multifunctional treatment systems. Some nanomaterials combine drug delivery with biological activity (Wang et al., 2025, Yang et al., 2023, Zang et al., 2025, Zhu et al., 2026). Bioactive glass nanoparticles and hydroxyapatite nanomaterials are similar to natural bone minerals. These materials can stimulate the activity of osteoblasts and promote bone regeneration (Wang et al., 2024a; Kou et al., 2021). Other systems integrate imaging agents. These platforms support treatment and diagnosis. Researchers are also studying the interaction between nanomaterials and the skeletal microenvironment. Bone tissue has a unique mineral composition and hierarchical structure. These characteristics create opportunities for targeted delivery strategies. Nanoparticles can be designed to bind firmly with hydroxyapatite crystals. This targeted strategy can improve the accumulation of drugs on the surface of bone remodeling. These advances highlight the potential of nanotechnology to overcome many limitations of traditional therapies. Nanomedicine provides new possibilities for improving the accuracy of treatment. This field continues to develop rapidly.

One of the most promising advances in the field of bone nanomedicine is the design of bone-targeted nanomaterials. These systems contain molecules that can specifically bind bone minerals (Li et al., 2026). Targeted ligands, such as bisphosphonate, tetracycline derivatives and acid oligopeptides, have a strong affinity for hydroxyapatite. When these ligands are connected to nanoparticles, they can guide the carrier to the skeletal tissue. Bone-targeted nanomaterials can deliver therapeutic drugs more effectively (You et al., 2025; Liang et al., 2025). This targeting mechanism can improve the accumulation of drugs in the bone remodeling area. Higher local drug concentrations can improve the treatment effect. At the same time, reducing the exposure of the whole body can minimize adverse reactions. This strategy solves one of the most critical challenges in the treatment of osteoporosis.

Research has further established the versatility of bone-targeting nanocarriers for the delivery of diverse therapeutic agents. These include anti-resorptive drugs, anabolic growth factors, and RNA-based therapies. This multifunctional capacity significantly broadens the therapeutic landscape. Moreover, nanoparticles can be engineered to release their payload in response to local pathological signals; for instance, shifts in pH or elevated oxidative stress within the diseased bone microenvironment can trigger spatiotemporally controlled drug release (Han et al., 2023; Kimura et al., 2021). A pivotal advantage lies in their potential to recalibrate bone homeostasis. By simultaneously engaging multiple cellular pathways, targeted nanomaterials can exert concerted effects: some systems inhibit osteoclast activity while promoting osteoblast maturation, whereas others modulate the interplay between inflammatory signaling and skeletal immunity. Such combined actions may cooperatively steer the system toward physiological bone remodeling. The rapid progress in bone-targeted nanotechnology is fostering convergence across disciplines—including materials science, pharmacology, and orthopedics—with continuous innovation poised to transform the management of osteoporosis.

This review aims to provide a comprehensive overview of recent advances in bone-targeting nanomaterials for osteoporosis therapy. We begin by discussing the biological basis of imbalanced bone remodeling, as understanding its cellular and molecular underpinnings is essential for developing targeted interventions. Subsequently, we summarize current strategies for bone-targeted drug delivery, with a focus on ligands that selectively bind to bone mineral components, which form the foundation of targeted nanomedicines. Various nanomaterial platforms, including lipid nanoparticles, polymeric carriers, and inorganic nanomaterials, are also introduced (Table 1, Section 5). The following section delves into how these nanomaterials regulate pivotal cellular processes in bone remodeling, such as osteoclast inhibition, osteoblast activation, and the modulation of bone cell signaling. Furthermore, we highlight emerging therapeutic modalities, exemplified by RNA nanomedicines and stimulus-responsive nanosystems, which represent a significant frontier in the field. The final part evaluates the persistent challenges in clinical translation, where issues concerning biosafety, targeting efficacy, and scalable manufacturing must be rigorously addressed; overcoming these hurdles is crucial for deploying nanomedicine-based therapies in the clinic. In summary, this review elucidates the potential of bone-targeting nanomaterials to reshape bone homeostasis and improve osteoporosis management. As this field continues to evolve rapidly, sustained interdisciplinary research is expected to yield novel strategies for safeguarding skeletal health.

**Table 1:** Comparative analysis of bone-seeking nanomaterial platforms.

Types	Representative Platforms	Principle	Efficiency	Safety/Clinical potential	Challenges
Lipid-Based Nanoparticles	Liposomes; SLNs	Dependent on surface modifications (such as bisphosphonates, polypeptides)	Flexible loading of hydrophilic& hydrophobic drugs; High biocompatibility; Membrane-like structure; Suitable for nucleic acid delivery	H	Long-term storage stability
Polymer Nanoparticles	PLGA; Polymeric micelles	Dependent on surface grafting of targeting ligands (such as Asp polypeptides and antibodies)	Tunable degradation; Sustained release; Easy surface functionalization	M	Inter-batch consistency Residual organic solvents; Coupling efficiency control of surface modification
Inorganic Nanomaterials	MSNs; HA	Conducive to ligand loading	Structural stability; Bone-mimetic composition; Sustained release; ion-mediated bioactivity; High loading capacity	L-M	The uniformity of large-scale production; The complexity of surface chemical modification; Ensure a balance between the long-term stability of the material in the body and its controllable degradation
Mixed Nanomaterials	Polymer-inorganic composite nanoparticle; Bioinspired nanocarriers	The polymer matrix typically governs controlled drug release and enables facile surface functionalization, while the inorganic component imparts enhanced structural stability and intrinsic osteoconductivity.	Combines strengths of multiple materials; Immune evasion; Enhanced precision; Dual functionality	L	Quality control; Standardized production processes (GMP)

Note: H-High, M-Middle, L-Low.

Importantly, the novelty of this review lies not simply in cataloguing bone-seeking nanocarriers, but in reframing the field from the perspective of restoring bone remodeling balance. Compared with prior reviews that mainly discuss material classes, targeting ligands, or isolated anabolic versus anti-resorptive strategies, the present review integrates osteoclast, osteoblast, osteocyte, and osteoimmune regulation within a single conceptual framework, while also highlighting emerging modalities such as RNA nanomedicine, exosome-inspired systems, stimulus-responsive platforms, and the translational barriers that govern clinical implementation.

## 2 Literature selection strategy

To provide a focused narrative overview, we conducted literature searches in PubMed, Web of Science, and Scopus for English-language articles published through March 2026. Search terms included combinations of “osteoporosis,” “bone-targeted drug delivery,” “bone-seeking nanomaterials,” “nanoparticles,” “osteoclast,” “osteoblast,” “osteocyte,” “osteoimmunology,” “RNA delivery,” and “exosome.” Priority was given to studies with direct relevance to bone-targeting strategies, modulation of bone remodeling, biomaterial design, and translational development, while seminal earlier reports were retained when they established key biological or nanotechnological concepts. Reference lists of selected papers were also manually screened to identify additional pertinent articles. Because the aim of this review is conceptual synthesis rather than formal meta-analysis, the final literature set was interpreted narratively with emphasis on mechanistic insight, platform characteristics, and translational significance.

## 3 Bone remodeling imbalance in osteoporosis

### 3.1 Physiological bone remodeling

**Fig. 1:**
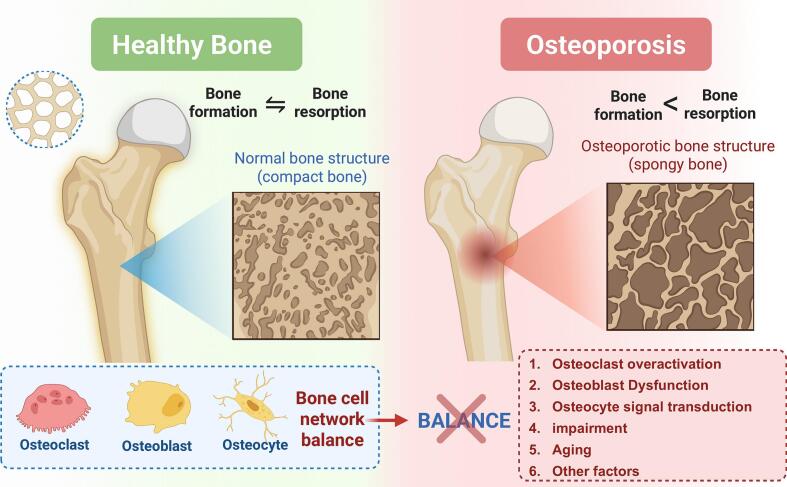
Imbalance of bone remodeling in healthy bone and osteoporosis. Healthy bone maintains skeletal homeostasis through a balanced bone cell network in which osteoclast-mediated bone resorption is tightly coupled to osteoblast-mediated bone formation, with osteocytes coordinating remodeling signals. In osteoporosis, this balance is disrupted by osteoclast overactivation, osteoblast dysfunction, impaired osteocyte signaling, aging, and other pathogenic factors, leading to reduced bone mass and deterioration of trabecular microarchitecture.

Bone is a dynamic organ that undergoes continuous remodeling, a lifelong biological process essential for renewing bone tissue and maintaining its structural integrity (Fig. 1). This process is orchestrated by specialized cells. Osteoclasts are responsible for resorbing aged or damaged bone tissue. Osteoblasts, in contrast, produce new bone matrix and facilitate its mineralization. The activities of these two cell types are coupled in a tightly regulated cycle, a concept known as osteoclast-osteoblast coupling (Chen et al., 2018; Zou et al., 2016). In healthy bone, the resorptive and formative phases are balanced, maintaining stable bone mass during adulthood. The bone remodeling cycle comprises sequential phases. It begins with the differentiation of osteoclast precursors into active osteoclasts. These cells attach to the bone surface and form sealed compartments called resorption lacunae, where they dissolve the mineralized matrix and degrade organic components, such as type I collagen (Xu et al., 2024). This resorption phase is transient. Osteoclasts subsequently undergo apoptosis or detach from the bone surface. Following this, osteoblast precursors are recruited to the remodeling site. These osteoblasts deposit osteoid, the organic bone matrix, which subsequently mineralizes with hydroxyapatite crystals. The newly formed bone tissue gradually restores the structural unit. Thereafter, the cycle enters a quiescent period until a new remodeling event is initiated.

The transition between these stages is regulated by a complex interplay of signaling pathways. Osteoclasts release coupling factors that recruit osteoblasts, a process aided by growth factors embedded within the bone matrix, such as transforming growth factor-β (TGF-β), insulin-like growth factor (IGF), and bone morphogenetic proteins (BMPs) (Zhou and Graves, 2022; Matsuo and Irie, 2008). Osteoblasts respond to these signals to initiate bone formation. The precise balance of these pathways ensures coordinated bone remodeling, which is fundamental to bone strength.

Osteocytes, the most abundant bone cells, play a central regulatory role in this process. Derived from osteoblasts entombed within the mineralized matrix, they form an extensive communication network via canaliculi. This network facilitates the exchange of biochemical signals throughout bone tissue (Sims and Martin, 2020; Ikebuchi et al., 2018). A key function of osteocytes is mechanosensation—the ability to sense mechanical load and transduce it into biochemical signals. For instance, mechanical stimulation inhibits the secretion of sclerostin, a protein that potently inhibits osteoblast activity. The consequent decrease in sclerostin levels promotes bone formation, illustrating how the osteocyte network helps bones adapt to mechanical stress (Xiang et al., 2025; Collins et al., 2024).

Consequently, osteocytes are pivotal coordinators of bone homeostasis. This physiological remodeling also supports systemic mineral homeostasis. The skeleton serves as a reservoir for calcium and phosphate. Osteoclast-mediated resorption releases these minerals into the circulation, while osteoblast-mediated formation stores them. Hormonal regulators like parathyroid hormone (PTH), vitamin D, and calcitonin coordinate these processes by influencing bone cell activity, thereby maintaining systemic mineral balance. Thus, bone remodeling is essential not only for skeletal integrity but also for metabolic regulation. The maintenance of healthy bone requires a precise equilibrium: osteoclastic resorption must match osteoblastic formation, and osteocytic signaling must remain intact. The disruption of any component can destabilize this balance, leading to conditions such as osteoporosis.

### 3.2 Cellular mechanism of osteoporosis

Osteoporosis arises from a disruption in the cellular balance of bone remodeling (Fig. 1). This imbalance is driven by multiple pathological mechanisms, with the overactivation of osteoclasts being a cardinal factor. In many osteoporotic conditions, both the enhanced differentiation and extended lifespan of mature osteoclasts lead to excessive bone resorption, causing bone degradation to outpace formation (Wang et al., 2023; Liu et al., 2022). The differentiation of osteoclasts—which originate from the monocyte-macrophage lineage—is stimulated by a suite of signals. Macrophage colony-stimulating factor (M-CSF) supports precursor survival, while receptor activator of nuclear factor-κB ligand (RANKL) signaling is pivotal for their maturation. This lineage is highly responsive to inflammatory stimuli, meaning chronic inflammation can markedly accelerate osteoclastogenesis. Factors such as aging, estrogen deficiency, and metabolic stress further exacerbate this process, leading to progressive erosion of the bone matrix.

Estrogen deficiency is a well-established driver of osteoclast activation, underpinning postmenopausal osteoporosis. Estrogen normally suppresses osteoclast differentiation and promotes their apoptosis. Its decline releases this protective brake, resulting in prolonged osteoclast survival and accelerated resorption, which explains the rapid bone loss observed in postmenopausal women. Concurrently, osteoporosis involves the impaired function of osteoblasts (Raisz, 2005; Yu and Wang, 2022). With aging, the differentiation capacity of osteoblasts declines due to waning osteogenic potential of mesenchymal stem cells (MSCs). Many MSCs undergo adipogenic rather than osteogenic differentiation, increasing bone marrow fat content. This reduction in osteoblast number and activity limits bone formation, tilting the remodeling balance toward resorption. At a molecular level, aging diminishes the activity of key transcription factors like Runx2 and Osterix. Furthermore, oxidative stress, through elevated reactive oxygen species (ROS), damages osteogenic signaling pathways, reducing matrix synthesis and mineralization.

Osteocyte dysfunction represents another critical mechanism (Cheng et al., 2022; Fischer and Haffner-Luntzer, 2022). With age, osteocytes undergo apoptosis, disrupting the lacunar-canalicular network and impairing the bone's mechanosensory capability. Additionally, aging osteocytes secrete higher levels of sclerostin, a potent inhibitor of osteoblast activity, thereby further suppressing bone formation. The age-related decline in physical activity reduces mechanical loading, diminishing the anabolic stimuli perceived by osteocytes and weakening adaptive bone formation.

Cellular senescence further fuels disease progression. Senescent osteoblasts and osteocytes adopt a senescence-associated secretory phenotype (SASP), releasing inflammatory cytokines and matrix-degrading enzymes that promote osteoclast differentiation (Yao et al., 2025). This creates a pro-resorptive local microenvironment, establishing a vicious cycle that accelerates bone loss.

In summary, the cellular pathology of osteoporosis entails three interrelated aberrations: (1) enhanced osteoclast activity; (2) diminished osteoblast function; and (3) impaired osteocyte signaling. Together, these changes compromise bone strength and predispose to fragility.

### 3.3 Molecular signaling pathways

**Fig. 2:**
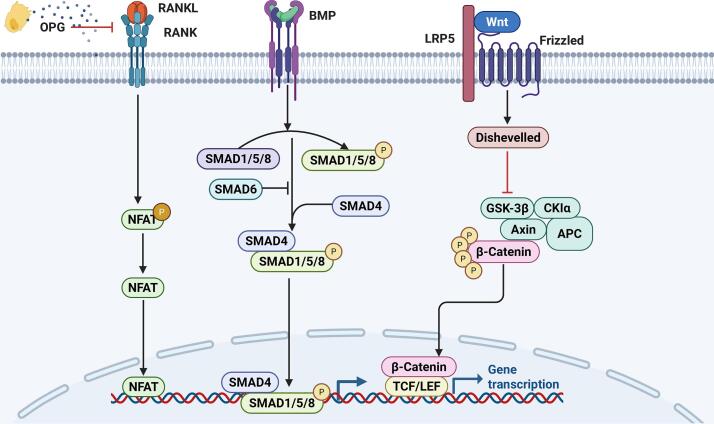
Major signaling pathways regulating bone remodeling in osteoporosis. Bone remodeling is tightly controlled by the OPG/RANKL/RANK, BMP/SMAD, and Wnt/β-catenin pathways, which coordinately regulate osteoclast differentiation, osteoblast activity, and downstream osteogenic gene transcription. Dysregulation of these interconnected signaling cascades contributes to excessive bone resorption and impaired bone formation in osteoporosis, highlighting their importance as therapeutic targets for bone-targeted nanomedicine.

Bone remodeling is governed by a complex, integrated network of molecular signals (Fig. 2). Among these, the RANKL-RANK-OPG axis is the pivotal regulator of osteoclast differentiation and activity (Monti et al., 2024; Sisay et al., 2017). RANKL, expressed by osteoblasts and osteocytes, binds to its receptor RANK on osteoclast precursors. This interaction activates key transcription factors such as NFATc1 and NF-κB, driving osteoclast maturation and bone resorption. Osteoprotegerin (OPG), a decoy receptor for RANKL, competitively inhibits this interaction by binding RANKL, thereby suppressing osteoclastogenesis (Daoussis et al., 2010; Liang et al., 2023). Healthy bone maintains a precise balance between RANKL and OPG. In osteoporosis, this balance is frequently shifted toward heightened RANKL signaling, leading to excessive osteoclast activity.

Bone formation is primarily regulated by the canonical Wnt/β-catenin signaling pathway (Wu et al., 2023; Wang et al., 2024b). Binding of Wnt ligands to receptors including LRP5/6 and Frizzled on osteoblast progenitors stabilizes cytoplasmic β-catenin, allowing its translocation to the nucleus where it activates a pro-osteogenic transcriptional program. This pathway is thus a potent stimulator of osteoblast differentiation and bone formation. Its critical role is underscored by human genetics, as mutations in *LRP5*significantly affect bone mineral density. The Wnt pathway is tightly regulated by endogenous inhibitors. Sclerostin (SOST) and Dickkopf-1 (DKK1) bind to LRP5/6, blocking Wnt activation (Rossini et al., 2013; Yang et al., 2025). Osteocytes are the primary source of sclerostin, whose secretion is upregulated under conditions of low mechanical strain. Elevated sclerostin levels, common in osteoporosis, potently inhibit osteoblast activity, contributing to reduced bone formation. The bone morphogenetic protein (BMP) pathway also plays a crucial anabolic role. As members of the TGF-β superfamily, BMPs (notably BMP-2 and BMP-7) signal through SMAD transcription factors in osteoprogenitor cells to promote osteoblast differentiation and matrix production. Attenuated BMP signaling is associated with impaired bone regeneration.

Inflammatory signaling pathways significantly impact bone homeostasis. Pro-inflammatory cytokines such as tumor necrosis factor-α (TNF-α), interleukin-1 (IL-1), and interleukin-6 (IL-6) promote osteoclastogenesis by enhancing RANKL expression and activating osteoclastogenic transcription factors (Fischer and Haffner-Luntzer, 2022; Heinrich et al., 2003). This nexus is the focus of osteoimmunology. Concurrently, chronic inflammation can suppress osteoblast function by inhibiting osteogenic differentiation and exacerbating oxidative stress. The aging-associated chronic, low-grade inflammation, termed “inflammaging,” is a key driver of age-related osteoporosis through these dual catabolic effects.

These major pathways do not operate in isolation but form a highly interconnected network. For instance, Wnt signaling can modulate RANKL expression, inflammatory cytokines influence BMP activity, and mechanical signals regulate sclerostin secretion. Osteoporosis can therefore be viewed as a consequence of dysfunction within this integrated signaling network, leading to a sustained imbalance between bone resorption and formation.

### 3.4 Enlightenment on therapeutic targets

Conventional osteoporosis therapies predominantly target a single component of the remodeling cascade: anti-resorptives inhibit osteoclast activity, whereas anabolics stimulate osteoblast function. However, these monotherapeutic approaches often fail to fully restore physiological bone remodeling balance. Effective long-term management may require simultaneously targeting multiple biological processes—curtailing excessive resorption while promoting formation—to achieve a net positive bone balance.

As discussed in Section 2.3, osteoclastogenesis is strongly driven by excessive RANKL signaling, whereas osteoblast activity is sustained by pro-osteogenic pathways such as Wnt/β-catenin. Accordingly, current therapeutic targets center on restraining pathologic resorption while restoring bone formation. Denosumab exemplifies the former strategy by neutralizing RANKL and thereby reducing osteoclast formation and bone resorption (Zhang et al., 2022; Sølling et al., 2026). On the anabolic side, inhibition of sclerostin relieves repression of Wnt signaling and promotes osteoblast differentiation and bone formation; romosozumab is a representative example that has shown significant gains in bone mineral density in clinical trials (Rosen, 2003; Palacios and Mejía, 2015).

Concurrently, achieving precise spatiotemporal drug action is critical. Targeted delivery systems that enhance drug accumulation at bone remodeling sites can maximize efficacy while minimizing systemic exposure (Chang et al., 2024). Bone-targeting nanomedicines are of particular interest, as they offer the potential to co-deliver agents that simultaneously regulate osteoclast and osteoblast activity at the lesion. Taken together, restoring physiological bone balance demands a comprehensive, network-based strategy. Future therapeutic innovation must consider the integrated remodeling circuit, paying concerted attention to intercellular communication, pathway crosstalk, and microenvironmental regulation. The ultimate goal is a holistic approach that sustains the delicate equilibrium between bone formation and resorption, thereby improving long-term skeletal outcomes.

## 4 Bone-targeting strategies for nanomedicine

### 4.1 Biological characteristics of bones as targets

Bone tissue possesses distinct structural and biochemical properties that render it an ideal target for nanomedicine. Its most prominent feature is a mineralized matrix rich in hydroxyapatite (HA), a crystalline compound of calcium and phosphate that provides structural rigidity (Thangavel and Elsen Selvam, 2022; El-Bahrawy et al., 2025). The exposed mineral surface offers abundant binding sites for molecules that interact with calcium ions, a property frequently exploited in drug delivery. Nanocarriers functionalized with calcium-chelating groups (e.g., phosphonates, carboxylates) can anchor to HA, promoting accumulation within bone tissue (Li et al., 2025a; Ye et al., 2020). This targeted accumulation can enhance local drug efficacy while reducing off-target distribution.

The skeletal vascular network is crucial for systemic delivery. Blood vessels permeate cortical and trabecular bone via specialized canals, delivering oxygen, nutrients, and circulating cells. This network also serves as a conduit for intravenously administered nanoparticles (Li et al., 2025a; Ye et al., 2020). Nanocarriers with prolonged circulation half-lives can leverage this vascular access to reach bone surfaces efficiently.

Bone is also dynamically remodeled. Osteoclasts resorb aged matrix, and osteoblasts deposit new bone within discrete bone remodeling units (BRUs). This process continuously exposes fresh, chemically reactive HA and collagen surfaces, which exhibit high affinity for targeting ligands (Liu et al., 2023; Samadi et al., 2022). Pathological states like osteoporosis, characterized by accelerated remodeling, further increase the availability of these binding sites, allowing nanocarriers to concentrate precisely where bone loss is most active.

The microstructure of bone, particularly the porous trabecular lattice, presents a vast surface area rich in HA binding sites. Nanoparticles accessing this space encounter numerous adhesion points. Furthermore, the mineralized matrix is a relatively stable biological compartment (Porter et al., 2009; Shadjou and Hasanzadeh, 2015). Unlike rapidly renewing soft tissues, nanoparticles bound to HA can achieve sustained local retention, prolonging therapeutic action—a significant advantage for chronic conditions like osteoporosis. In summary, the unique composition, vascularity, dynamic surface, and microstructure of bone collectively establish a supportive environment for targeted nanomedicine.

### 4.2 Bone-seeking ligands

The development of bone-seeking ligands is central to targeted nanomedicine. These molecules, conjugated to nanocarriers, enable selective binding to bone mineral or specific cell types, drastically improving therapeutic precision. Bisphosphonates (BPs) are the most established ligands. Containing two phosphonate groups, BPs exhibit a high affinity for HA calcium ions (Lam et al., 2007; Abbasi, 2011). Nanocarriers decorated with BP ligands efficiently localize to sites of active remodeling and newly exposed mineral surfaces, forming a reliable foundation for bone-targeted therapies. Tetracycline derivatives also possess an innate affinity for mineralized tissue. Modified to eliminate antibiotic activity while retaining calcium-binding capacity, these molecules can guide nanoparticles to areas of active mineralization.

Aspartic acid-rich peptides offer a versatile, biocompatible strategy. Sequences containing multiple aspartate residues present negatively charged carboxyl groups that interact strongly with HA calcium ions, enabling firm nanoparticle adhesion (Abbasi, 2012; Gano et al., 2007). Certain peptides can be engineered for cell-specific targeting, such as binding preferentially to osteoblasts, enabling direct drug delivery to anabolic cells.

Emerging ligands include aptamers (short nucleic acids selected to bind bone-specific proteins) and antibody fragments targeting receptors on osteoclasts, osteoblasts, or other microenvironment constituents (Ferreira et al., 2012). These promise high cellular specificity. Additionally, multifunctional strategies are being explored, where a single nanoparticle carries both a mineral-binding ligand (e.g., BP) and a cell-specific targeting molecule (e.g., peptide or antibody), enhancing both bone homing and cellular precision.

A more critical comparison indicates that no single ligand class is universally optimal. Bisphosphonate-based ligands generally provide the strongest and most reproducible hydroxyapatite affinity, which favors skeletal accumulation, but they may show limited selectivity among different bone surfaces and can introduce pharmacologic effects of their own. In contrast, aspartic acid-rich peptides offer better molecular tunability and potentially lower off-target toxicity, yet their binding strength and in vivo stability may be more sensitive to sequence design and proteolytic degradation. Aptamers and antibody-derived ligands are attractive when cell-level precision is required, particularly for distinguishing osteoclast-, osteoblast-, or immune-associated targets, but this gain in specificity is offset by greater manufacturing complexity, higher cost, and more demanding stability control. Therefore, ligand selection should be guided by the intended therapeutic objective, namely broad mineral targeting, cell-selective delivery, or balanced dual-targeting, rather than by hydroxyapatite affinity alone.

### 4.3 Design considerations for bone-targeted nanocarriers

The efficacy of bone-targeted nanocarriers hinges on meticulous optimization of their physicochemical properties, which govern circulation, targeting, and drug release. Size is a critical parameter. Nanoparticles sized between ∼50–200 nm typically achieve an optimal balance: they are small enough to avoid rapid splenic/liver sequestration yet large enough to evade immediate renal clearance, allowing sufficient time to reach bone tissue (Moradi et al., 2025; Parks et al., 1993). Circulation Time must be sufficiently long for nanoparticles to reach and bind bone. PEGylation—the surface grafting of polyethylene glycol (PEG) chains—is a standard method to reduce opsonization and macrophage uptake, thereby prolonging circulation half-life (Bhandari et al., 2015).

Ligand Density and Presentation on the nanoparticle surface directly influence binding strength to HA. While high density can enhance affinity, it may also cause nanoparticle aggregation or alter biodistribution. An optimal density must be empirically determined to balance strong binding with colloidal stability. Surface Chemistry, including charge and hydrophobicity, affects protein adsorption and stability in blood. Near-neutral or slightly negative surfaces generally minimize non-specific protein binding and improve biocompatibility. The conjugation chemistry must also ensure ligands remain stably attached during systemic travel. Drug Release Kinetics are equally crucial (Hu et al., 2015; Wen et al., 2022). Ideal carriers retain their payload during circulation and release it in a controlled manner at the target site. This can be achieved through slow diffusion or, more sophisticatedly, by incorporating stimuli-responsive elements (e.g., pH- or enzyme-sensitive linkers) that trigger release specifically within the bone remodeling microenvironment. Therefore, successful bone-targeted nanocarrier design requires the holistic integration of insights from bone biology, materials science, and pharmacology. Continuous refinement of these parameters is driving progress toward more effective therapies for osteoporosis and other skeletal disorders.

## 5 Bone-seeking nanomaterial platforms

### 5.1 Lipid-based nanoparticles

**Fig. 3:**
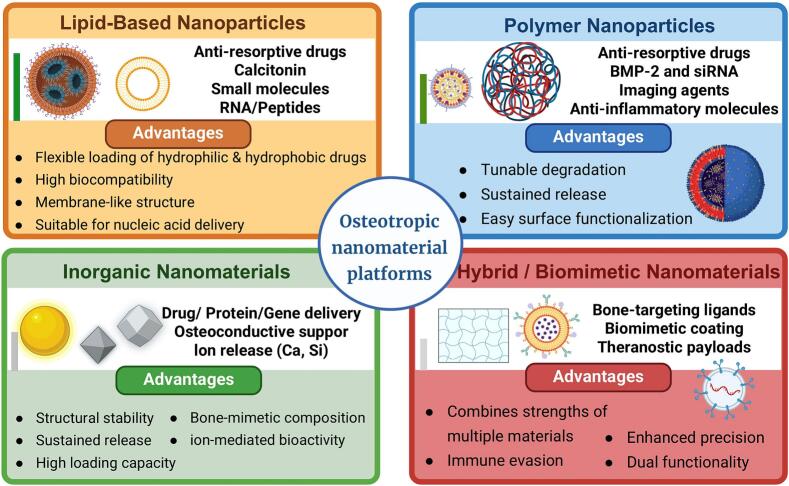
Major osteotropic nanomaterial platforms for osteoporosis therapy. Osteotropic nanomaterials used for osteoporosis treatment can be broadly classified into lipid-based nanoparticles, polymer nanoparticles, inorganic nanomaterials, and hybrid/biomimetic nanoplatforms, each with distinct cargo-loading properties, targeting strategies, and therapeutic functions. The entity item on the left side of each plot indicates the safety/clinical potential of the platforms, with the higher height and greener indicating high or middle degree. On the contrary, the gray and shorter one indicates low degree.

Lipid-based nanoparticles, encompassing liposomes and solid lipid nanoparticles (SLNs), are among the most established and versatile platforms in nanomedicine (Fig. 3). Their structural core, composed of lipid bilayers that mimic natural cell membranes, grants them inherent biocompatibility, low toxicity, and favorable biodistribution, making them a prevalent choice for therapeutic nanocarrier design (Hallan et al., 2022; Rahmani et al., 2025).

Liposomes are spherical vesicles with an aqueous core enclosed by a phospholipid bilayer. This unique architecture allows for the co-encapsulation of both hydrophilic drugs (within the aqueous interior) and hydrophobic compounds (embedded within the lipid membrane), significantly enhancing the versatility of drug delivery (González-Fernández et al., 2015).

Solid Lipid Nanoparticles (SLNs), typically comprising surfactant-stabilized solid or semi-solid lipid cores, offer superior physical stability compared to traditional liposomes. They are particularly valued for their ability to protect sensitive cargoes, such as RNA therapeutics and peptides, from degradation (Alam et al., 2023; Joun et al., 2022). The proven success of lipid nanoparticles in nucleic acid delivery has further spurred their investigation for bone disease therapy.

For bone-targeting applications, the surface of these lipid nanoparticles is commonly functionalized with ligands such as bisphosphonates or aspartic acid-rich peptides. This modification enables selective binding to hydroxyapatite in bone tissue, thereby increasing drug accumulation at skeletal sites and reducing off-target distribution (Takeda et al., 2016; Wang et al., 2024c). A key advantage of lipid nanocarriers is their high drug-loading capacity and flexibility. They can encapsulate a wide range of therapeutics, including anti-resorptive agents (e.g., bisphosphonates, calcitonin), anabolic compounds, and anti-inflammatory drugs, supporting diverse strategies to simultaneously inhibit osteoclast activity and stimulate osteoblast function.

In summary, lipid-based nanomaterials provide a multifunctional platform for bone-targeted drug delivery. Their excellent biocompatibility, adaptable drug-loading modalities, and ease of surface engineering render them ideal candidates. Ongoing advancements in lipid nanoparticle technology continue to expand their potential for effective osteoporosis treatment.

### 5.2 Polymer nanoparticles

Polymeric nanoparticles constitute a versatile and highly tunable class of nanocarriers for bone-targeted drug delivery (Fig. 3). Composed of biodegradable polymers, they form stable nanostructures capable of encapsulating diverse therapeutic agents. A key advantage is the precise control over their chemical properties, which allows for customization of drug release kinetics, degradation rates, and surface functionality (Briffault et al., 2024; Chen et al., 2021). Among the most prevalent materials is poly(lactic-*co*-glycolic acid) (PLGA), a copolymer with regulatory approval for medical use. PLGA nanoparticles degrade in vivo into lactic and glycolic acid, metabolites that are safely cleared by the body, underpinning their excellent safety profile. The polymer matrix protects encapsulated cargo (e.g., anti-resorptive drugs, osteogenic factors, nucleic acids) from premature degradation, while its gradual hydrolysis enables sustained drug release over extended periods, maintaining therapeutic concentrations at the target site (Hlukhaniuk et al., 2024).

Polymeric micelles, formed by the self-assembly of amphiphilic block copolymers in aqueous solutions, represent another important type. They possess a hydrophobic core for solubilizing poorly water-soluble drugs and a hydrophilic shell that ensures colloidal stability in biological fluids. Their typically small size contributes to favorable circulation profiles. Dendrimers are a third class, characterized by a highly branched, monodisperse architecture with a multitude of surface functional groups. This precise structure permits extensive surface modification and ligand conjugation (e.g., with bone-targeting moieties or imaging agents), enabling high drug-loading capacity and controlled molecular interactions (Ortega-Oller et al., 2015; Zhang et al., 2025a).

For bone-targeting applications, the surface of polymeric nanoparticles can be functionalized with ligands such as bisphosphonates or aspartic acid-rich peptides to direct them to bone mineral. Furthermore, surface engineering with coatings like polyethylene glycol (PEG) reduces immune clearance, prolongs circulation, and enhances the likelihood of bone deposition. The inherent multifunctionality of polymeric systems supports the development of advanced theranostic platforms that combine, for instance, drug delivery with imaging capabilities or the co-delivery of agents targeting different aspects of bone remodeling (e.g., anti-inflammatory and anabolic) (Bagchi et al., 2014; Bender et al., 2024). Thus, polymeric nanoparticles offer a highly flexible platform due to their tunable degradation, controllable drug release, and versatile surface chemistry. These attributes make them indispensable for constructing sophisticated, bone-specific therapeutic systems for osteoporosis.

### 5.3 Inorganic nanomaterials

Inorganic nanomaterials constitute a versatile platform for bone-targeted therapy, offering unique structural, compositional, and functional advantages (Fig. 3). Their inherent stability, mechanical robustness, and, for many, biomimetic composition (resembling natural bone mineral) make them particularly suitable for applications requiring both drug delivery and bone regeneration (Zhang et al., 2024; Zhu and Li, 2023).

Mesoporous silica nanoparticles (MSNs) are a widely investigated class. They possess a highly ordered porous structure with an exceptionally high surface area, providing ample space for loading therapeutic agents such as drugs, proteins, or nucleic acids. Precise control over pore size and surface chemistry allows for customizable drug release profiles. For bone targeting, MSNs are often surface-functionalized with ligands like bisphosphonates to enable selective binding to bone hydroxyapatite, guiding the carrier to the desired site (Zhou et al., 2024).

Hydroxyapatite (HA) nanoparticles are of particular interest due to their chemical and structural identity with the native mineral phase of bone. This biomimicry allows HA nanoparticles to readily integrate with skeletal tissue. They serve as excellent carriers for osteogenic factors while also exhibiting strong osteoconductivity—the ability to promote osteoblast adhesion, proliferation, and new bone formation (Zorrón et al., 2024). Thus, they function dually as a drug delivery vehicle and a supportive scaffold for bone regeneration.

Bioactive glass nanoparticles represent a third promising category. Composed primarily of silica, calcium, and phosphate, they interact dynamically with physiological fluids. Upon dissolution, they release ions (e.g., Ca^2+^, Si^4+^) that stimulate osteogenic signaling pathways, enhancing osteoblast differentiation and mineralization. This osteoinductive capability, coupled with the potential to promote angiogenesis, provides a powerful bioactive stimulus alongside drug delivery (Chen et al., 2023). Therefore, inorganic nanomaterials offer a compelling combination of structural durability for reliable drug carriage and biological activity that actively supports bone healing. This dual functionality renders them highly attractive for developing advanced, bone-targeted therapeutic platforms.

### 5.4 Mixed nanomaterials

Researchers are continually advancing the frontier of nanomedicine by developing hybrid (or composite) nanomaterials that integrate distinct material classes into a single, multifunctional therapeutic platform (Fig. 3). These systems are engineered to synergize the advantages of their components—such as the tailored degradability of polymers with the structural robustness of inorganic materials—while mitigating individual limitations, often resulting in enhanced targeting efficiency and therapeutic potency (Wu et al., 2026; Hertz and Bruce, 2007).

A prominent design is the polymer-inorganic composite nanoparticle, which combines biodegradable polymers (e.g., PLGA) with inorganic materials like hydroxyapatite (HA) or silica. In such constructs, the polymer matrix typically governs controlled drug release and enables facile surface functionalization, while the inorganic component imparts enhanced structural stability and intrinsic osteoconductivity. This synergy creates versatile carriers capable of concurrently supporting drug delivery and bone regeneration.

Bioinspired nanocarriers constitute another innovative direction. These systems incorporate biomimetic elements, such as bone-specific peptides, proteins, or extracellular matrix components, to closely emulate the natural bone microenvironment. This biomimicry not only improves biocompatibility but also actively promotes osteoblast adhesion and tissue integration, facilitating more effective bone repair.

An especially promising strategy is cell membrane coating. In this approach, nanoparticles are cloaked with membranes derived from source cells (e.g., macrophages or mesenchymal stem cells). The resulting vesicles inherit the parent cell's surface proteins and identity markers, which can confer abilities like immune evasion and active homing. For instance, macrophage membrane-coated nanoparticles can be directed to inflammatory sites associated with active bone remodeling, enabling precise drug delivery to the pathological skeletal niche (Miyazaki, 2013; Moosavifar et al., 2022).

The intrinsic multifunctionality of hybrid nanomaterials supports advanced theranostic applications. A single particle can be designed to deliver therapeutic cargo, modulate the local immune response, provide osteogenic signals, and even carry imaging agents for real-time monitoring. Targeting accuracy is further refined by combining multiple mechanisms, such as coupling mineral-binding ligands (e.g., bisphosphonates) with bioinspired surface motifs, creating a synergistic effect that maximizes delivery efficiency to bone tissue.

In summary, the development of sophisticated hybrid nanomaterials reflects the growing complexity and ambition of bone-targeted nanomedicine. These integrated platforms, capable of addressing multiple facets of skeletal pathology, hold significant promise for creating future therapies that effectively restore bone remodeling balance in osteoporosis.

Viewed comparatively, the major nanomaterial platforms display distinct trade-offs rather than a simple hierarchy of advancement. Lipid-based carriers are often advantageous for biocompatibility and nucleic acid loading, but their structural softness can limit long-term colloidal stability and promote premature cargo leakage. Polymeric systems provide broader control over release kinetics and surface engineering, yet formulation heterogeneity, residual solvent concerns, and scale-up reproducibility may complicate translation. Inorganic nanomaterials contribute mechanical robustness and, in some cases, intrinsic osteoconductive or osteoinductive activity, but their slower degradation and potential tissue persistence raise additional biosafety considerations. Hybrid platforms are conceptually attractive because they combine multiple functions within a single construct; however, the same structural sophistication often increases manufacturing burden, analytical complexity, and regulatory uncertainty. Accordingly, the most suitable platform is not necessarily the most multifunctional one, but the one whose material properties, payload compatibility, and translational feasibility are best matched to the intended clinical scenario.

## 6 Nanomaterial-mediated modulation of bone remodeling

### 6.1 Nanotherapy targeting osteoclast activity

**Fig. 4:**
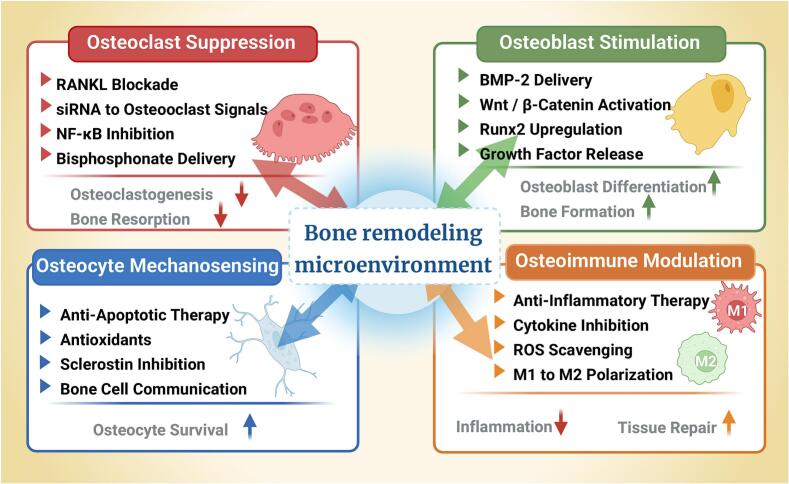
Nano-enabled regulation of the bone remodeling microenvironment in osteoporosis. Bone-targeted nanotherapeutics can restore remodeling balance by simultaneously suppressing osteoclastogenesis through RANKL blockade, siRNA delivery, NF-κB inhibition, and bisphosphonate-based anti-resorptive strategies, while promoting osteoblast differentiation and bone formation via BMP-2 delivery, Wnt/β-catenin activation, Runx2 upregulation, and growth factor release. In parallel, these nanosystems improve osteocyte survival and mechanosensing through anti-apoptotic and antioxidant interventions, and remodel the osteoimmune niche by reducing inflammatory cytokines, scavenging ROS, and promoting macrophage polarization from the M1 to the M2 phenotype.

Excessive osteoclast activity is a central driver of bone loss in osteoporosis (Fig. 4). Osteoclasts, derived from the monocyte-macrophage lineage, are uniquely responsible for resorbing the mineralized bone matrix. In healthy remodeling, osteoclastic bone resorption is tightly coupled to osteoblast-mediated bone formation. Osteoporosis disrupts this equilibrium, leading to a pathologic state where enhanced osteoclast differentiation and activity predominate. Consequently, inhibiting osteoclast function represents a crucial therapeutic strategy for restoring skeletal integrity.

A major nanotherapeutic focus is the RANKL-RANK axis because it lies upstream of osteoclast differentiation and activation (De Leon-Oliva et al., 2023; van Dam et al., 2020). Nanoparticle delivery systems can be engineered to transport agents that disrupt this pathway, such as RANKL-neutralizing antibodies, small interfering RNA (siRNA), or small-molecule inhibitors. Targeted delivery enhances drug concentration at bone surfaces while minimizing systemic exposure.

Another critical regulatory node is the transcription factor NF-κB, a key mediator of osteoclastogenesis. NF-κB activation stimulates the expression of genes essential for osteoclast differentiation and survival; its inhibition can potently suppress osteoclast activity (Di Nisio et al., 2015; Kaneko et al., 2025). Nanocarriers have been developed to deliver NF-κB inhibitors directly to bone tissue, thereby attenuating inflammatory signaling and blocking the transcriptional program driving osteoclast formation.

Beyond signaling pathway inhibition, nanoplatforms can directly deliver anti-resorptive agents, such as bisphosphonates, to impair osteoclast function. These targeted nanoparticles accumulate preferentially on active bone remodeling surfaces rich in osteoclasts, achieving a more potent local effect than systemic administration.

Experimental studies substantiate the promise of these approaches. For instance, liposomal carriers loaded with bisphosphonates selectively accumulate in bone and inhibit osteoclast activity. Similarly, polymeric nanoparticles have been used to deliver siRNA targeting RANKL, effectively suppressing osteoclast differentiation through gene silencing (Raje et al., 2019). These strategies underscore the advantage of nanomedicine in achieving precise, localized modulation of bone resorption.

Altogether, nanotherapeutics aimed at osteoclasts—through targeted inhibition of differentiation, RANKL signaling, or NF-κB activation—constitute a promising avenue for reducing pathological bone degradation and are a vital component of the targeted nanomedicine arsenal against osteoporosis.

### 6.2 Nanomaterials that promote the differentiation of osteoblasts

While inhibiting bone resorption is crucial, effective osteoporosis treatment must also stimulate osteoblast activity to enhance bone formation (Fig. 4). Osteoblasts, derived from mesenchymal stem cells (MSCs), are responsible for synthesizing the bone matrix and initiating its mineralization. Impaired osteoblast function is a key contributor to bone fragility, driving the development of nanomaterial platforms designed to enhance osteogenic differentiation. A primary strategy involves the targeted delivery of osteoinductive proteins. Bone morphogenetic protein-2 (BMP-2), a member of the TGF-β superfamily pivotal for skeletal development, is a major candidate (Haversath et al., 2012; Tateiwa et al., 2021). BMP-2 activates intracellular SMAD signaling to drive MSC commitment toward the osteoblastic lineage. Nanoparticle carriers can protect encapsulated BMP-2 from proteolytic degradation during circulation and facilitate its sustained release at the bone surface, thereby enhancing osteogenic activity and new bone formation.

Nanocarriers are also being developed to deliver anabolic agents that engage the osteogenic pathways outlined in Section 2.3, particularly BMP/SMAD and Wnt/β-catenin signaling. By improving the stability and bioavailability of these molecules, nanoparticle-based delivery can enhance osteoblast differentiation and matrix mineralization. Additional strategies include delivery of Runx2-inducing molecules and pro-regenerative growth factors, such as vascular endothelial growth factor and fibroblast growth factors, to support osteoblast maturation, angiogenesis, and microenvironmental repair (Ziros et al., 2008; Zhang and Cho, 2023).

Beyond molecular delivery, certain inorganic nanomaterials (e.g., hydroxyapatite, bioactive glass) provide dual functionality. They act as bioactive scaffolds that promote osteoblast adhesion and proliferation, while their ionic dissolution products (e.g., Ca^2+^, Si^4+^) can directly activate osteogenic signaling pathways. This combination of drug delivery and structural bioactivity synergistically enhances the overall regenerative outcome.

### 6.3 Nanomaterials regulating osteocytes and mechanosensing

Recent research has underscored the pivotal role of osteocytes—the most abundant and long-lived bone cells—in orchestrating bone remodeling (Fig. 4). Differentiated from mature osteoblasts and embedded within the mineralized matrix, osteocytes form an extensive communication network via canaliculi. This network is essential for mechanosensation, the process by which osteocytes perceive mechanical loads (e.g., from physical activity) and transduce these physical signals into biochemical cues that regulate osteoblast and osteoclast activity (Katsianou et al., 2025; Dalle Carbonare et al., 2012). Impairment of this mechanosensory function contributes to the dysregulated bone loss observed in osteoporosis.

A key pathological feature is osteocyte apoptosis, which increases with aging and metabolic stress. The loss of osteocytes disrupts intercellular communication within the bone microenvironment, weakening the coordination between bone-forming and bone-resorbing cells. Consequently, nanomaterials designed to deliver anti-apoptotic agents or antioxidants to osteocytes may help preserve this cellular network and maintain remodeling balance (Jonason et al., 2009; Komori, 2008). Osteocytes are also the primary source of sclerostin, a potent inhibitor of the Wnt/β-catenin signaling pathway in osteoblasts. Elevated sclerostin levels, commonly found in osteoporotic bone, suppress bone formation. Therefore, nanotherapeutic strategies aim to downregulate sclerostin signaling. Nanocarriers can deliver molecules that silence sclerostin gene expression or block its interaction with Wnt co-receptors, thereby antagonizing its inhibitory effect and promoting osteogenesis (Vishal et al., 2016). Targeted delivery ensures these modulators reach osteocyte-rich bone regions.

An emerging frontier involves developing mechano-responsive nanomaterials that release therapeutic payloads in response to mechanical stress within bone tissue, mimicking and augmenting native mechanotransduction pathways. Although still in early stages, this approach holds promise for creating bioactive implants or injectable depots that are activated by patient movement.

### 6.4 Bone immunomodulation nanomaterials

Bone remodeling is not an isolated process but is deeply intertwined with the immune system, a crosstalk studied in the field of osteoimmunology (Fig. 4). Immune cells—including macrophages, T lymphocytes, and dendritic cells—secrete cytokines that critically influence the activity of both osteoclasts and osteoblasts. Chronic inflammatory states often disrupt this balance, accelerating bone loss. This understanding of immune-bone interactions has unveiled new therapeutic avenues for intervention (Zhu and Li, 2023; Chu et al., 2025).

A key mechanism involves macrophage polarization. Macrophages can adopt distinct functional phenotypes: the pro-inflammatory M1 phenotype, which secretes cytokines that promote osteoclastogenesis and bone resorption, and the anti-inflammatory, pro-repair M2 phenotype, which supports tissue healing and osteogenesis. The dynamic balance between these phenotypes significantly impacts bone remodeling outcomes. Nanomaterials offer a powerful tool to modulate this balance within bone tissue. Engineered nanoparticles can deliver agents that suppress pro-inflammatory signaling and promote a shift from M1 to M2 polarization. This reprogramming can reduce the local levels of osteoclastogenic cytokines while fostering an environment conducive to osteoblast differentiation and bone formation (Wu et al., 2026; Chen et al., 2025). Furthermore, nanocarriers can be designed for the targeted delivery of anti-inflammatory drugs, antioxidants, or cytokine inhibitors directly to the inflammatory bone microenvironment, maximizing local efficacy while minimizing systemic side effects.

The surface properties of nanomaterials themselves can impart immunomodulatory functions. A prominent example is the use of cell-membrane coatings derived from immune cells. Nanoparticles cloaked with such membranes can evade immune clearance and actively engage with specific inflammatory pathways, enhancing their targeting to diseased bone sites. By mitigating chronic inflammation, immunomodulatory nanoplatforms remove a major barrier to effective bone regeneration, allowing osteoblasts to function more efficiently. Thus, strategic immune regulation concurrently supports bone anabolism and tissue repair. The convergence of nanomedicine and osteoimmunology represents a promising frontier in osteoporosis research. Nanocarriers capable of directing macrophage polarization, tuning inflammatory signaling, and modulating immune-bone crosstalk provide novel and sophisticated treatment possibilities. These strategies move beyond targeting bone cells alone to actively reshape the broader microenvironment that governs skeletal health.

In summary, nanomaterial-mediated regulation of bone remodeling offers multi-faceted intervention opportunities: inhibiting osteoclast activity curbs excessive resorption; enhancing osteoblast differentiation promotes bone formation; preserving osteocyte function maintains the mechanosensory network; and modulating immune responses stabilizes the bone microenvironment. Together, these integrated strategies underscore the significant potential of bone-targeted nanomedicine for the treatment of osteoporosis.

## 7 Advanced therapeutic nanoplatforms

### 7.1 Gene and RNA nanomedicine

**Fig. 5:**
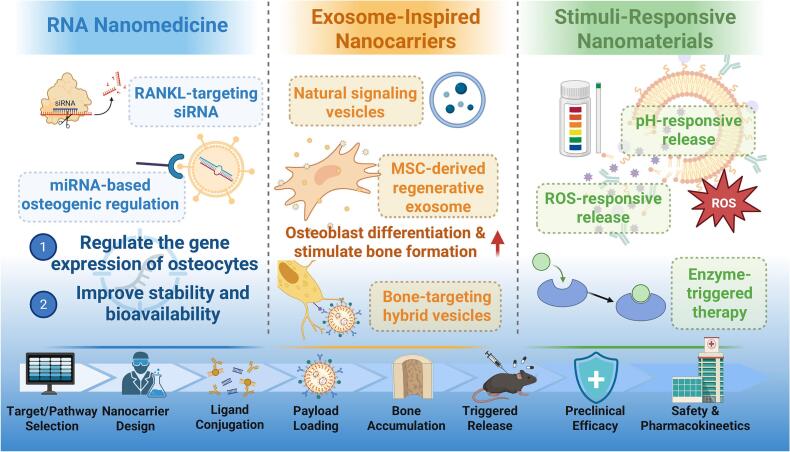
Emerging nanotherapeutic platforms and translational workflow for osteoporosis treatment. Advanced osteotropic nanomedicine strategies, including RNA nanomedicine, exosome-inspired nanocarriers, and stimuli-responsive nanomaterials, enable gene regulation, regenerative signaling delivery, and microenvironment-responsive drug release to enhance bone targeting and therapeutic precision. Their development toward clinical application follows a stepwise pathway from target selection and nanocarrier design to ligand conjugation, payload loading, bone accumulation, triggered release, preclinical validation, and safety and pharmacokinetic evaluation.

Recent advances in gene and RNA-based therapeutics have unveiled novel avenues to modulate bone remodeling in osteoporosis at the genetic level (Fig. 5). This approach centers on delivering nucleic acids—such as small interfering RNA (siRNA), microRNA (miRNA), and other regulatory oligonucleotides—to precisely control gene expression in bone cells. Nanocarriers are indispensable in this field, as they protect these vulnerable nucleic acids from enzymatic degradation in biological fluids and facilitate their delivery into target cells (Cui et al., 2022b; Seong et al., 2023).

A widely investigated strategy employs siRNA targeting RANKL, a key cytokine driving osteoclast differentiation. Nanoparticles designed to deliver RANKL-specific siRNA to bone tissue can achieve post-transcriptional gene silencing, reducing RANKL production, inhibiting osteoclastogenesis, and thereby limiting pathological bone resorption. Compared to conventional pharmacological inhibition, nanoparticle-mediated siRNA delivery offers distinct advantages. Targeted carriers accumulate at bone remodeling sites with high RANKL expression, enhancing local therapeutic efficacy. Furthermore, the nanocarrier shields the siRNA from degradation, significantly improving its stability and bioavailability (Lee et al., 2024). Various platforms, including lipid nanoparticles, polymeric vectors, and dendrimers, have shown promise for efficient siRNA delivery by promoting cellular uptake and facilitating endosomal escape—a critical step for effective gene silencing (Wang et al., 2025a).

MicroRNAs (miRNAs), endogenous regulators of gene expression, represent another powerful tool. Specific miRNAs are known to regulate osteogenic differentiation and bone formation; some promote osteoblast maturation, while others inhibit osteoclast activity. Consequently, researchers are developing nanocarriers to deliver miRNA mimics (to restore deficient osteogenic signals) or miRNA inhibitors (antagomiRs, to suppress pathogenic miRNAs) to bone cells. For instance, delivering osteogenic miRNAs that activate master transcription factors like Runx2 and Osterix can enhance bone formation and accelerate regeneration.

### 7.2 Exosome-inspired nanocarriers

Exosome-inspired nanocarriers represent a promising frontier in bone-targeted nanomedicine, bridging synthetic design with natural biological communication (Fig. 5). Exosomes are nanoscale extracellular vesicles naturally secreted by various cell types, carrying a cargo of proteins, lipids, and nucleic acids that mediate intercellular signaling and regulate diverse physiological processes (Lavrador et al., 2018; Zou et al., 2023). Their intrinsic role as nature's delivery vehicles has inspired their exploration as therapeutic nanocarriers.

Among potential sources, exosomes derived from mesenchymal stem cells (MSCs) are of particular interest for bone repair. MSC-exosomes harbor a rich payload of osteogenic microRNAs, growth factors, and regulatory proteins that can modulate bone cell behavior. Experimental studies demonstrate that these vesicles enhance osteoblast differentiation, stimulate bone formation—often by activating pathways like Wnt/β-catenin—and can mitigate inflammation in bone defect models, highlighting their multifaceted regenerative potential. A significant advantage of exosomes is their high biocompatibility and low immunogenicity, owing to their endogenous origin. Their lipid bilayer membrane protects the encapsulated cargo and facilitates cellular uptake, while their natural surface proteins may aid in crossing biological barriers and targeting specific cells. Despite these benefits, the clinical translation of natural exosomes faces hurdles, including challenges in scalable production, purification, and batch-to-batch variability, which can affect therapeutic consistency (Tylawsky et al., 2023; Nadar et al., 2017). Consequently, researchers are developing exosome-mimetic nanoparticles that recapitulate the advantageous features of natural vesicles using synthetic, more controllable platforms.

These bioinspired systems often combine synthetic nanomaterial cores with bioactive membrane coatings derived from source cells (e.g., MSCs or immune cells). This hybrid design mimics the structure and function of exosomes, providing a bionic surface for improved biocompatibility, immune evasion, and targeted interactions. Furthermore, they can be engineered for enhanced functionality, such as by integrating bone-targeting ligands (e.g., bisphosphonates) to improve skeletal homing, or by packaging specific therapeutic molecules.

Researchers are constantly exploring exosome nanocarriers that target bones by integrating bone-bound ligands. These hybrid systems combine the advantages of natural vesicles and synthetic nanoparticles. Such platforms are expected to accurately deliver regeneration signals to skeletal tissue. Therefore, nanocarriers inspired by exosome bodies are expected to become a bridge between nanotechnology and cell therapy. The continuous progress of vesicle engineering and bionic design is expected to enhance its application potential in bone regeneration and osteoporosis treatment.

### 7.3 Stimuli-responsive bone nanomaterials

A cutting-edge strategy in skeletal nanomedicine involves the development of stimuli-responsive nanomaterials capable of releasing their therapeutic payload in response to specific signals within the local bone microenvironment (Fig. 5). This approach enables spatiotemporally controlled drug delivery, enhancing treatment precision and minimizing off-target effects. Pathological conditions like osteoporosis are characterized by altered biochemical profiles at bone remodeling sites, including shifts in pH, elevated oxidative stress, and specific enzyme activities (Al Thaher et al., 2021; Xie et al., 2025). Nanomaterials can be engineered to detect and react to these changes, undergoing structural transformations that trigger drug release precisely where and when it is needed.

pH-responsive systems exploit the acidic microenvironment created by osteoclasts during bone resorption. Osteoclasts secrete protons to dissolve hydroxyapatite, locally lowering the pH. Nanocarriers incorporating acid-labile bonds or components remain stable at physiological pH but degrade and release their cargo (e.g., anti-resorptive agents) in this acidic niche, allowing direct inhibition of osteoclasts at active resorption pits. Reactive oxygen species (ROS)-responsive nanomaterials target the elevated oxidative stress prevalent in aged or inflamed bone. Excessive ROS contribute to osteoblast dysfunction and osteocyte apoptosis (Wang et al., 2025b; Ming et al., 2024). Nanoparticles designed with ROS-cleavable links (e.g., thioketal bonds) can deliver antioxidants or other drugs specifically to these high-ROS regions, providing targeted cytoprotection or therapy.

Enzyme-responsive strategies utilize enzymes upregulated in bone pathology as triggers. For instance, proteases (e.g., matrix metalloproteinases) and tartrate-resistant acid phosphatase (TRAP), which are highly active during osteoclast-mediated resorption, can be harnessed to cleave specific peptide sequences on nanocarriers, resulting in localized drug release.

The integration of stimuli-responsive mechanisms offers significant advantages: it ensures drugs remain inert during circulation, activating only at the disease site, thereby maximizing efficacy and safety. Furthermore, these systems align with the principles of precision medicine by adapting to the unique biochemical signature of the pathological microenvironment. A single, multifunctional nanoparticle can combine bone-targeting ligands, environmental sensors, and therapeutic components, creating a powerful tool to regulate the complex bone remodeling process. Continued innovation in this field is poised to yield more accurate and effective treatments for osteoporosis and other bone disorders.

Importantly, these advanced modalities also differ in translational maturity. Gene- and RNA-based systems offer high mechanistic precision and the possibility of directly reprogramming pathogenic pathways, but their performance remains strongly dependent on nuclease protection, cellular uptake, and efficient endosomal escape. Exosome-inspired platforms may better mimic endogenous intercellular communication and exhibit favorable biointerfacing properties, yet variability in source material, purification, and batch definition remains a major constraint. Stimuli-responsive nanomaterials provide superior spatiotemporal control in principle, but their therapeutic reliability depends on whether the triggering signal is sufficiently strong, stable, and disease-specific in vivo. Taken together, more sophisticated design does not automatically translate into greater clinical utility; in some settings, a simpler and more reproducible bone-targeted platform may offer a more realistic path toward therapeutic application.

## 8 Future perspectives

### 8.1 Toward precision nanomedicine

Future research in bone nanomedicine will likely focus on advancing precision nanomedicine approaches tailored to the individual's specific disease biology (Wu et al., 2025, Wu et al., 2025, Yuan, 2025). Nanotechnology provides a powerful toolkit to achieve this by enabling the design of carriers that respond to patient-specific pathological signatures, such as distinct patterns of bone remodeling activity, inflammatory signals, and oxidative stress. Precision nanocarriers could be engineered to release their therapeutic payload only upon encountering these disease-associated cues within bone tissue. The integration of diagnostic imaging with therapeutic delivery—creating theranostic platforms that combine treatment and monitoring within a single nanostructure—further supports this personalized paradigm (Liang et al., 2025; Cheng and Wang, 2024). Such strategies allow for treatment plans to be dynamically adjusted based on the unique pathophysiological profile of each patient.

### 8.2 Development of multifunctional nano-platforms

A key direction is the development of sophisticated multifunctional nanoplatforms that integrate several therapeutic actions into a single system. These platforms could simultaneously regulate osteoclast activity, stimulate osteoblast differentiation, and modulate local inflammation. By combining bone-targeting ligands, drug delivery modules, and stimuli-responsive mechanisms, they can achieve targeted accumulation and context-dependent drug release. Furthermore, platforms incorporating inorganic components (e.g., hydroxyapatite for osteoconduction) with polymeric carriers for molecule delivery can provide both biological signals and structural support for bone regeneration (Li et al., 2022; Sun et al., 2023). This comprehensive, multi-target strategy holds promise for producing more robust therapeutic outcomes than single-target therapies.

### 8.3 AI-guided rational design

The rapid advancement of artificial intelligence (AI) and machine learning (ML) offers transformative opportunities for nanocarrier design. AI algorithms can analyze vast datasets describing nanoparticle properties, biological interactions, and therapeutic outcomes to identify design principles that optimize targeting, efficacy, and safety. ML models can predict how parameters like size, surface chemistry, and ligand composition influence biodistribution and bone targeting (Xiong et al., 2024). Such computational tools can also optimize drug loading and release kinetics. By modeling nanoparticle-biological system interactions in silico, researchers can accelerate the development cycle and improve the rational design of next-generation bone-targeted nanomaterials.

### 8.4 Convergence with regenerative medicine

Future therapies may converge nanomedicine with regenerative biomaterials to enhance bone repair. Nanoparticles can be embedded within biomaterial scaffolds (e.g., hydrogels, 3D-printed matrices) that provide structural support for new bone formation. These hybrid systems allow for the sustained, localized release of osteogenic drugs as the scaffold degrades, combining molecular therapy with guided tissue regeneration. This integrated approach is particularly promising for treating severe bone defects and osteoporotic fractures, merging the strengths of advanced drug delivery with regenerative engineering.

### 8.5 Translational, manufacturing, and regulatory barriers

Despite encouraging preclinical efficacy, translation of bone-targeted nanomedicines remains constrained by the absence of a universally harmonized regulatory pathway for nanopharmaceuticals. In practice, most candidates are still evaluated within existing drug, biologic, or combination-product frameworks, which places particular emphasis on defining product-specific critical quality attributes, orthogonal analytical characterization, sterility control, impurity profiling, and pharmacokinetic comparability after manufacturing changes (Souto et al., 2024; De Jong et al., 2022; Đorđević et al., 2022). For osteotropic systems, these requirements are further complicated by multicomponent architectures in which ligand density, particle size distribution, surface charge, cargo loading, release kinetics, and protein-corona formation can all influence biodistribution and bone-targeting performance. Accordingly, regulatory translation will require early alignment between formulation design, chemistry-manufacturing-control strategies, and nonclinical testing rather than treating these steps as sequentially independent processes (Souto et al., 2024; De Jong et al., 2022; Đorđević et al., 2022).

Large-scale manufacturing presents an additional bottleneck. Many nanosystems that are reproducible at laboratory scale become difficult to control during scale-up because small variations in mixing rate, solvent exchange, purification, lyophilization, or sterilization can alter particle morphology, colloidal stability, and batch-to-batch consistency (Đorđević et al., 2022; Shen et al., 2024). This issue may be particularly relevant for hybrid or ligand-decorated bone-targeted platforms, where minor changes in surface composition can substantially affect hydroxyapatite affinity and in vivo fate. At the same time, long-term safety cannot be inferred from short-term efficacy studies alone. Although inorganic nanomaterials such as mesoporous silica, bioactive glass, and certain metal-based nanoparticles provide structural stability and osteogenic activity, slowly degradable or non-degradable fractions may persist in the reticuloendothelial system or other organs, raising concerns regarding chronic accumulation, delayed inflammatory responses, and cumulative toxicity (Saker et al., 2024; Yu et al., 2024). These uncertainties are magnified by the limitations of current animal models. Ovariectomized rodents and other preclinical models have been indispensable for mechanistic studies, but they do not fully reproduce human cortical osteonal remodeling, age-associated marrow alterations, lifelong comorbidity burden, or the complex immune-vascular features of the osteoporotic bone microenvironment (Koh et al., 2024). As a result, targeting efficiency, residence time, and safety profiles observed in small animals may overestimate performance in human skeletal tissue. Future translation will therefore benefit from integrating aged animal models, large-animal studies, and humanized or ex vivo bone microenvironment platforms into the preclinical evaluation pipeline (Shen et al., 2024; Koh et al., 2024).

### 8.6 Pharmaceutics-focused development considerations

From a pharmaceutics perspective, successful osteotropic nanomedicines must be developed as robust drug products rather than only as proof-of-concept carriers. This requires early definition of the intended product presentation, such as ready-to-use injectable dispersion, concentrated intermediate, or reconstitutable dry formulation, because that decision directly affects excipient selection, pH, osmolality, residual solvent control, sterilization strategy, and container–closure compatibility. For nucleic acid-loaded or hybrid nanosystems in particular, buffer composition, ionic strength, cryoprotectants or lyoprotectants, and downstream drying conditions can markedly influence particle size distribution, encapsulation efficiency, aggregation tendency, reconstitution behavior, and retention of biological activity after storage. Accordingly, quality-by-design principles are especially relevant in this field, as critical material attributes and critical process parameters jointly determine clinically meaningful critical quality attributes such as size, polydispersity, zeta potential, drug loading, release profile, sterility assurance, and shelf stability (U.S. Food and Drug Administration, 2018; European Medicines Agency, 2025; Clogston et al., 2024; Zhang et al., 2025b; VandenBerg et al., 2025; Arte et al., 2025).

Pharmacokinetic assessment should likewise move beyond nominal bone accumulation alone. Particle size, surface chemistry, ligand density, and protein-corona evolution can reshape systemic clearance, marrow exposure, skeletal residence time, and off-target uptake by the liver or spleen, thereby altering both efficacy and safety. For this reason, formulation optimization and scale-up should be linked to route-specific PK evaluation, including circulation half-life, area under the curve, apparent volume of distribution, bone-to-organ exposure ratios, and the relationship between release kinetics and pharmacodynamic response. In practical terms, formulations that preserve colloidal stability during manufacturing, frozen or lyophilized storage, transport, sterilization, and reconstitution while maintaining reproducible PK behavior may offer greater translational value than structurally elaborate systems with fragile manufacturability (European Medicines Agency, 2025; Clogston et al., 2024; Zhang et al., 2025b; VandenBerg et al., 2025; Arte et al., 2025; Peralta-Cuevas et al., 2026; Maheshwari et al., 2025).

## 9 Conclusions

Despite remarkable progress, the clinical translation of bone-targeted nanodrugs still faces substantial barriers that extend beyond proof-of-concept efficacy. A central challenge is that nanomedicines are generally regulated through existing pharmaceutical frameworks rather than through a unified nano-specific approval pathway, making robust definition of critical quality attributes, validated analytical methods, and manufacturing comparability essential for approval (Souto et al., 2024; De Jong et al., 2022; Đorđević et al., 2022). These requirements become particularly demanding for bone-targeted systems because particle size, ligand density, surface chemistry, and release behavior jointly determine both skeletal accumulation and systemic safety. In parallel, scalable manufacturing remains difficult: process-dependent variations introduced during mixing, purification, sterilization, or storage can compromise batch reproducibility and alter biological performance (Đorđević et al., 2022; Shen et al., 2024). Long-term biosafety also requires deeper investigation, especially for slowly degradable or non-degradable inorganic nanomaterials that may accumulate outside bone and produce delayed toxicological effects after repeated dosing (Saker et al., 2024; Yu et al., 2024). Finally, the translational value of current preclinical models remains limited, since commonly used rodent models do not fully recapitulate the cortical structure, remodeling dynamics, marrow composition, and immune-vascular microenvironment of human osteoporotic bone (Koh et al., 2024; Wu et al., 2024). Addressing these issues will require coordinated advances in regulatory science, manufacturing standardization, chronic toxicity assessment, and clinically relevant model systems. From a pharmaceutics standpoint, formulation robustness, storage stability, scalable sterile manufacture, and interpretable pharmacokinetic behavior should be treated as core design objectives rather than downstream optimization steps. Equally important, future success will depend on pharmaceutics-oriented optimization of dosage form, storage stability, scalable manufacturing, and PK reproducibility, because these attributes ultimately determine whether bone-targeted nanotherapeutics can be translated into clinically usable osteoporosis products (Arte et al., 2025; Peralta-Cuevas et al., 2026; Maheshwari et al., 2025; Wu et al., 2024; Cheng et al., 2017; Li et al., 2025b; Zhong et al., 2023; Ackun-Farmmer et al., 2022).

Beyond targeting, nanoparticle systems allow for the precise modulation of bone cell activities. Nanocarriers can be engineered to deliver agents that inhibit osteoclast-mediated resorption, stimulate osteoblast differentiation and bone formation, regulate osteocyte signaling, or modulate the inflammatory microenvironment associated with bone loss (Cheng et al., 2017). This capacity to simultaneously influence multiple cellular pathways represents a critical advantage, as effective osteoporosis treatment requires both curtailing excessive resorption and promoting new bone formation. Targeted delivery enhances local drug concentration while minimizing systemic exposure, thereby improving therapeutic efficiency.

Recent progress in RNA delivery, exosome-inspired systems, and stimuli-responsive nanomaterials has expanded the field beyond passive targeting toward programmable and microenvironment-responsive intervention. In parallel, bioactive inorganic components such as hydroxyapatite and bioactive glass offer the added advantage of supporting osteogenesis while serving as drug carriers (Li et al., 2025b). Collectively, these advances position bone-targeted nanomedicine as a platform for coordinated regulation of resorption, formation, osteocyte signaling, and osteoimmune crosstalk rather than simple single-pathway inhibition.

To move from promising preclinical prototypes to clinically deployable therapies, several future priorities deserve particular emphasis. First, studies should adopt more clinically relevant benchmarking standards, including direct comparison with approved osteoporosis drugs, route-specific pharmacokinetic and pharmacodynamic evaluation, bone-to-off-target exposure ratios, repeat-dose safety, and storage-to-use stability. Second, platform simplification should be prioritized over excessive structural complexity, because carriers with clearly defined composition, scalable manufacture, and reproducible critical quality attributes are more likely to achieve regulatory translation than highly multifunctional but analytically opaque systems. Third, preclinical validation should move toward models that better reflect human disease heterogeneity, including aged animals, fracture-associated osteoporosis settings, and bone microenvironment models incorporating marrow, vascular, and immune components (Chang et al., 2024; Koh et al., 2024; Arte et al., 2025; Peralta-Cuevas et al., 2026; Maheshwari et al., 2025; Wu et al., 2024; Cheng et al., 2017; Li et al., 2025b; Zhong et al., 2023; Ackun-Farmmer et al., 2022).

Finally, future development should be guided by realistic therapeutic positioning. The most achievable near-term applications may not be universal treatment of all osteoporosis subtypes, but rather selected scenarios in which bone-targeted delivery offers a clear advantage, such as high-turnover osteoporosis, impaired osteoporotic fracture healing, or combination therapy for patients with insufficient response or intolerance to existing agents. In summary, bone-targeted nanomaterials provide a compelling framework for coordinating anti-resorptive, anabolic, osteocyte-protective, and immunomodulatory actions within the skeletal microenvironment. However, future success will depend less on adding further complexity and more on achieving a balanced integration of biological rationale, pharmaceutics robustness, safety, and translational manufacturability. Prioritizing reproducible formulations, clinically relevant validation, and clearly defined therapeutic niches will be essential for converting this concept from an academically attractive strategy into practical products for osteoporosis management (Souto et al., 2024; De Jong et al., 2022; Đorđević et al., 2022; Shen et al., 2024; Saker et al., 2024; Yu et al., 2024; Koh et al., 2024; U.S. Food and Drug Administration, 2018; European Medicines Agency, 2025; Clogston et al., 2024; Zhang et al., 2025b; VandenBerg et al., 2025; Arte et al., 2025; Peralta-Cuevas et al., 2026; Maheshwari et al., 2025; Wu et al., 2024; Cheng et al., 2017; Li et al., 2025b; Zhong et al., 2023; Ackun-Farmmer et al., 2022).

## CRediT authorship contribution statement

**Fei Wang:** Writing – original draft. **Feng Liang:** Investigation. **Xiaonan Zhou:** Investigation. **Yuanyuan Ding:** Investigation. **Mingzhe Wu:** Formal analysis. **Nan Li:** Writing – review & editing. **Wei Yan:** Writing – review & editing, Supervision, Project administration.

## Funding

This research did not receive any specific grant from funding agencies in the public, commercial, or not-for-profit sectors.

## Declaration of competing interest

Not applicable.

## Data Availability

Data will be made available on request.

## References

[bb0005] Abbasi I.A. (2011). Studies on 177Lu-labeled methylene diphosphonate as potential bone-seeking radiopharmaceutical for bone pain palliation. Nucl. Med. Biol..

[bb0010] Abbasi I.A. (2012). Preliminary studies on (177)Lu-labeled sodium pyrophosphate (177Lu-PYP) as a potential bone-seeking radiopharmaceutical for bone pain palliation. Nucl. Med. Biol..

[bb0015] Ackun-Farmmer M.A., Xiao B., Newman M.R., Benoit D.S.W. (2022). Macrophage depletion increases target specificity of bone-targeted nanoparticles. J. Biomed. Mater. Res. A.

[bb0020] Al Thaher Y., Alotaibi H.F., Yang L., Prokopovich P. (2021). PMMA bone cement containing long releasing silica-based chlorhexidine nanocarriers. PLoS One.

[bb0025] Alam S.B., Wang F., Qian H., Kulka M. (2023). Apolipoprotein C3 facilitates internalization of cationic lipid nanoparticles into bone marrow-derived mouse mast cells. Sci. Rep..

[bb0030] Anam A.K., Insogna K. (2021). Update on osteoporosis screening and management. Med. Clin. North Am..

[bb0035] Arte K.S., Chen M., Patil C.D., Huang Y., Qu L., Zhou Q. (2025). Recent advances in drying and development of solid formulations for stable mRNA and siRNA lipid nanoparticles. J. Pharm. Sci..

[bb0040] Bagchi A., Meka S.R., Rao B.N., Chatterjee K. (2014). Perovskite ceramic nanoparticles in polymer composites for augmenting bone tissue regeneration. Nanotechnology.

[bb0045] Bender E.C., Sircar A.J., Taubenfeld E.K., Suggs L.J. (2024). Modulating lipid-polymer nanoparticles' physicochemical properties to alter macrophage uptake. ACS Biomater. Sci. Eng..

[bb0050] Bhandari K.H., Asghar W., Newa M., Jamali F., Doschak M.R. (2015). Evaluation of bone targeting salmon calcitonin analogues in rats developing osteoporosis and adjuvant arthritis. Curr. Drug Deliv..

[bb0055] Black D.M., Rosen C.J. (2016). Clinical practice. Postmenopausal osteoporosis. N. Engl. J. Med..

[bb0060] Briffault E., Reyes R., Garcia-Garcia P., Rouco H., Diaz-Gomez L., Arnau M.R., Evora C., Diaz-Rodriguez P., Delgado A. (2024). SFRP1-silencing GapmeR-loaded lipid-polymer hybrid nanoparticles for bone regeneration in osteoporosis: effect of dosing and targeting strategy. Int. J. Nanomedicine.

[bb0065] Capozzi A., Lello S., Pontecorvi A. (2014). The inhibition of RANK-ligand in the management of postmenopausal osteoporosis and related fractures: the role of denosumab. Gynecol. Endocrinol..

[bb0070] Chang Z., Chen D., Peng J., Liu R., Li B., Kang J., Guo L., Hou R., Xu X., Lee M., Zhang X. (2024). Bone-targeted supramolecular nanoagonist assembled by accurate ratiometric herbal-derived therapeutics for osteoporosis reversal. Nano Lett..

[bb0075] Chen X., Wang Z., Duan N., Zhu G., Schwarz E.M., Xie C. (2018). Osteoblast-osteoclast interactions. Connect. Tissue Res..

[bb0080] Chen D., Liu Y., Zhang Z., Liu Z., Fang X., He S., Wu C. (2021). NIR-II fluorescence imaging reveals bone marrow retention of small polymer nanoparticles. Nano Lett..

[bb0085] Chen X., Li H., Ma Y., Jiang Y. (2023). Calcium phosphate-based nanomaterials: preparation, multifunction, and application for bone tissue engineering. Molecules.

[bb0090] Chen L., Zhu J., Ge N., Liu Y., Yan Z., Liu G., Li Y., Wang Y., Wu G., Qiu T., Dai H., Han J., Guo C. (2025). A biodegradable magnesium alloy promotes subperiosteal osteogenesis via interleukin-10-dependent macrophage immunomodulation. Biomaterials.

[bb0095] Cheng R., Wang S. (2024). Cell-mediated nanoparticle delivery systems: towards precision nanomedicine. Drug Deliv. Transl. Res..

[bb0100] Cheng H., Chawla A., Yang Y., Li Y., Zhang J., Jang H.L., Khademhosseini A. (2017). Development of nanomaterials for bone-targeted drug delivery. Drug Discov. Today.

[bb0105] Cheng C.H., Chen L.R., Chen K.H. (2022). Osteoporosis due to hormone imbalance: an overview of the effects of estrogen deficiency and glucocorticoid overuse on bone turnover. Int. J. Mol. Sci..

[bb0110] Chu X., Mi B., Xiong Y., Wang R., Liu T., Hu L., Yan C., Zeng R., Lin J., Fu H., Liu G., Zhang K., Bian L. (2025). Bioactive nanocomposite hydrogel enhances postoperative immunotherapy and bone reconstruction for osteosarcoma treatment. Biomaterials.

[bb0115] Clogston J.D., Foss W., Harris D., Oberoi H., Pan J., Pu E., Torrico Guzmán E.A., Walter K., Brown S., Lim Soo P. (2024). Current state of nanomedicine drug products: an industry perspective. J. Pharm. Sci..

[bb0120] Collins J.M., Lang A., Parisi C., Moharrer Y., Nijsure M.P., Thomas Kim J.H., Ahmed S., Szeto G.L., Qin L., Gottardi R., Dyment N.A., Nowlan N.C., Boerckel J.D. (2024). YAP and TAZ couple osteoblast precursor mobilization to angiogenesis and mechanoregulation in murine bone development. Dev. Cell.

[bb0125] Cortet B., Guañabens N., Brandi M.L., Siggelkow H. (2024). Similarities and differences between European guidelines for the management of postmenopausal osteoporosis. Arch. Osteoporos..

[bb0130] Cosman F. (2009). Treatment of osteoporosis and prevention of new fractures: role of intravenously administered bisphosphonates. Endocr. Pract..

[bb0135] Cosman F. (2014). Anabolic and antiresorptive therapy for osteoporosis: combination and sequential approaches. Curr. Osteoporos. Rep..

[bb0140] Cui J., Shibata Y., Zhu T., Zhou J., Zhang J. (2022). Osteocytes in bone aging: advances, challenges, and future perspectives. Ageing Res. Rev..

[bb0145] Cui Y., Guo Y., Kong L., Shi J., Liu P., Li R., Geng Y., Gao W., Zhang Z., Fu D. (2022). A bone-targeted engineered exosome platform delivering siRNA to treat osteoporosis. Bioact. Mater..

[bb0150] Dalle Carbonare L., Innamorati G., Valenti M.T. (2012). Transcription factor Runx2 and its application to bone tissue engineering. Stem Cell Rev. Rep..

[bb0155] Daoussis D., Andonopoulos A.P., Liossis S.N. (2010). Wnt pathway and IL-17: novel regulators of joint remodeling in rheumatic diseases. Looking beyond the RANK-RANKL-OPG axis. Semin. Arthritis Rheum..

[bb0160] De Jong W.H., Geertsma R.E., Borchard G. (2022). Regulatory safety evaluation of nanomedical products: key issues to refine. Drug Deliv. Transl. Res..

[bb0165] De Leon-Oliva D., Barrena-Blázquez S., Jiménez-Álvarez L., Fraile-Martinez O., García-Montero C., López-González L., Torres-Carranza D., García-Puente L.M., Carranza S.T., Álvarez-Mon M., Álvarez-Mon M., Diaz R., Ortega M.A. (2023). The RANK-RANKL-OPG system: a multifaceted regulator of homeostasis, immunity, and cancer. Medicina (Kaunas).

[bb0170] Di Nisio C., Zizzari V.L., Zara S., Falconi M., Teti G., Tetè G., Nori A., Zavaglia V., Cataldi A. (2015). RANK/RANKL/OPG signaling pathways in necrotic jaw bone from bisphosphonate-treated subjects. Eur. J. Histochem..

[bb0175] Đorđević S., Gonzalez M.M., Conejos-Sánchez I., Carreira B., Pozzi S., Acúrcio R.C., Satchi-Fainaro R., Florindo H.F., Vicent M.J. (2022). Current hurdles to the translation of nanomedicines from bench to the clinic. Drug Deliv. Transl. Res..

[bb0180] El-Bahrawy N.R., Elmekawy A., Salem M.L., Morsy R. (2025). Gelatin-hydroxyapatite-based hybrid composites: enhanced mechanical and biological characteristics through biomaterials integration for bone tissue engineering applications. Int. J. Biol. Macromol..

[bb0185] European Medicines Agency (2025).

[bb0190] Ferreira S., Dormehl I., Botelho M.F. (2012). Radiopharmaceuticals for bone metastasis therapy and beyond: a voyage from the past to the present and a look to the future. Cancer Biother. Radiopharm..

[bb0195] Fischer V., Haffner-Luntzer M. (2022). Interaction between bone and immune cells: implications for postmenopausal osteoporosis. Semin. Cell Dev. Biol..

[bb0200] Fleseriu M. (2018). Recombinant growth hormone treatment, osteoporosis and fractures, more complicated than it seems!. Endocrine.

[bb0205] Gano L., Marques F., Campello M.P., Balbina M., Lacerda S., Santos I. (2007). Radiolanthanide complexes with tetraazamacrocycles bearing methylphosphonate pendant arms as bone seeking agents. Q. J. Nucl. Med. Mol. Imaging.

[bb0210] Gao S., Zhao Y. (2023). Quality of life in postmenopausal women with osteoporosis: a systematic review and meta-analysis. Qual. Life Res..

[bb0215] González-Fernández Y., Imbuluzqueta E., Patiño-García A., Blanco-Prieto M.J. (2015). Antitumoral-lipid-based nanoparticles: a platform for future application in osteosarcoma therapy. Curr. Pharm. Des..

[bb0220] Hallan S.S., Amirian J., Brangule A., Bandere D. (2022). Lipid-based nano-sized cargos as a promising strategy in bone complications: a review. Nanomaterials (Basel).

[bb0225] Han S., Xue L., Wei Y., Yong T., Jia W., Qi Y., Luo Y., Liang J., Wen J., Bie N., Liang H., Liu Q., Ding Z., Yang X., Gan L., Huang Z., Chen X., Zhang B. (2023). Bone lesion-derived extracellular vesicles fuel prometastatic cascades in hepatocellular carcinoma by transferring ALKBH5-targeting miR-3190-5p. Adv. Sci. (Weinh.).

[bb0230] Haversath M., Catelas I., Li X., Tassemeier T., Jäger M. (2012). PGE₂ and BMP-2 in bone and cartilage metabolism: 2 intertwining pathways. Can. J. Physiol. Pharmacol..

[bb0235] Heinrich P.C., Behrmann I., Haan S., Hermanns H.M., Müller-Newen G., Schaper F. (2003). Principles of interleukin (IL)-6-type cytokine signalling and its regulation. Biochem. J..

[bb0240] Hertz A., Bruce I.J. (2007). Inorganic materials for bone repair or replacement applications. Nanomedicine (London).

[bb0245] Hlukhaniuk A., Świętek M., Patsula V., Hodan J., Janoušková O., Bystrianský L., Brož A., Malić M., Zasońska B., Tokarz W., Bačáková L., Horák D. (2024). Poly(ε-caprolactone)-based composites modified with polymer-grafted magnetic nanoparticles and L-ascorbic acid for bone tissue engineering. J Biomed Mater Res B Appl Biomater.

[bb0250] Hu Y., Li J., Zhu X., Li Y., Zhang S., Chen X., Gao Y., Li F. (2015). 17β-estradiol-loaded PEGlyated upconversion nanoparticles as a bone-targeted drug nanocarrier. ACS Appl. Mater. Interfaces.

[bb0255] Huang X., Li S., Lu W., Xiong L. (2022). Metformin activates Wnt/β-catenin for the treatment of diabetic osteoporosis. BMC Endocr. Disord..

[bib771] Huang Q., Liao J., Li J., Gu Z., Zhang X., Sun M., Lu J. (2025). Curcumin-loaded ceria nanoenzymes for dual-action suppression of inflammation and alleviation of oxidative damage in the treatment of acute lung injury. Chin. Chem. Lett..

[bb0260] Ikebuchi Y., Aoki S., Honma M., Hayashi M., Sugamori Y., Khan M., Kariya Y., Kato G., Tabata Y., Penninger J.M., Udagawa N., Aoki K., Suzuki H. (2018). Coupling of bone resorption and formation by RANKL reverse signalling. Nature.

[bb0265] Jiang Z., Qi G., He X., Yu Y., Cao Y., Zhang C., Zou W., Yuan H. (2024). Ferroptosis in osteocytes as a target for protection against postmenopausal osteoporosis. Adv. Sci. (Weinh.).

[bb0270] Jonason J.H., Xiao G., Zhang M., Xing L., Chen D. (2009). Post-translational regulation of Runx2 in bone and cartilage. J. Dent. Res..

[bb0275] Joun I., Nixdorf S., Deng W. (2022). Advances in lipid-based nanocarriers for breast cancer metastasis treatment. Front. Med. Technol..

[bb0280] Kaneko T., Yari S., Kikuta J., Omatsu Y., Seno S., Kikuchi S., Sato K., Fujii K., Sudo T., Hasegawa T., Furuta K., Guo Q., Ibrahim S.H., Muraoka K., Okada Y., Kubota Y., Okuzaki D., Kobayashi Y., Kumanogoh A., Udagawa N., Nagasawa T., Penninger J.M., Ishii M. (2025). The RANK/RANKL axis controls vascular dynamics in the bone marrow. Proc. Natl. Acad. Sci. USA.

[bb0285] Katsianou M.A., Gargalionis A.N., Papavassiliou K.A., Margoni A., Papavassiliou A.G., Basdra E.K. (2025). The critical role of transcription factor RUNX2 in bone mechanobiology. Cells.

[bb0290] Kimura T., Bosakova M., Nonaka Y., Hruba E., Yasuda K., Futakawa S., Kubota T., Fafilek B., Gregor T., Abraham S.P., Gomolkova R., Belaskova S., Pesl M., Csukasi F., Duran I., Fujiwara M., Kavkova M., Zikmund T., Kaiser J., Buchtova M., Krakow D., Nakamura Y., Ozono K., Krejci P. (2021). An RNA aptamer restores defective bone growth in FGFR3-related skeletal dysplasia in mice. Sci. Transl. Med..

[bb0295] Koh N.Y.Y., Miszkiewicz J.J., Fac M.L., Wee N.K.Y., Sims N.A. (2024). Preclinical rodent models for human bone disease, including a focus on cortical bone. Endocr. Rev..

[bb0300] Komori T. (2008). Regulation of bone development and maintenance by Runx2. Front. Biosci..

[bb0305] Kou L., Jiang X., Lin X., Huang H., Wang J., Yao Q., Chen R. (2021). Matrix metalloproteinase inspired therapeutic strategies for bone diseases. Curr. Pharm. Biotechnol..

[bb0310] Lam M.G., de Klerk J.M., van Rijk P.P., Zonnenberg B.A. (2007). Bone seeking radiopharmaceuticals for palliation of pain in cancer patients with osseous metastases. Anti Cancer Agents Med. Chem..

[bb0315] Lavrador P., Gaspar V.M., Mano J.F. (2018). Stimuli-responsive nanocarriers for delivery of bone therapeutics - barriers and progresses. J. Control. Release.

[bb0320] Lee S.Y., Kim S.J., Park K.H., Lee G., Oh Y., Ryu J.H., Huh Y.H. (2024). Differential but complementary roles of HIF-1α and HIF-2α in the regulation of bone homeostasis. Commun. Biol..

[bb0325] Lerner U.H. (2006). Bone remodeling in post-menopausal osteoporosis. J. Dent. Res..

[bb0330] Li J., Chen X., Lu L., Yu X. (2020). The relationship between bone marrow adipose tissue and bone metabolism in postmenopausal osteoporosis. Cytokine Growth Factor Rev..

[bib768] Li Z., Yu H., Wang Z., Duan H., Li M., Liao J., Yang L. (2025). Recent advances in nanotechnology for repairing spinal cord injuries. Biomaterials.

[bb0335] Li Z., Zhang W., Zhang Z., Gao H., Qin Y. (2022). Cancer bone metastases and nanotechnology-based treatment strategies. Expert Opin. Drug Deliv..

[bib767] Li Xuemei, Li Zhe, Liu Jing, Hongbin Zang, Yang Ying, Tan Xiaolin, Liao Jun, Ce Wang (2026). Engineering stem cell-based nanotherapeutics to overcome myocardial ischemia-reperfusion injury. Biomaterials.

[bb0340] Li Y., Li X., Zhu L., Liu T., Huang L. (2025). Chitosan-based biomaterials for bone tissue engineering. Int. J. Biol. Macromol..

[bb0345] Li L., Rong G., Gao X., Cheng Y., Sun Z., Cai X., Xiao J. (2025). Bone-targeted fluoropeptide nanoparticle inhibits NF-κB signaling to treat osteosarcoma and tumor-induced bone destruction. Adv. Sci. (Weinh.).

[bb0350] Liang Q.L., Xu H.G., Yu L., Ding M.R., Li Y.T., Qi G.F., Zhang K., Wang L., Wang H., Cui X. (2023). Binding-induced fibrillogenesis peptide inhibits RANKL-mediated osteoclast activation against osteoporosis. Biomaterials.

[bb0355] Liang K., Zhao L., Zhang S., Zheng L., Zhang Z., Wang S., Chen J., Xu W., Wang W., Yang H., Song C., Qiu P., Zhao C., Fang W., Zhu J., Fan S., Liu Z., Tang R., Zhao Y., Fang X. (2025). Spatiotemporal-adaptive nanotherapeutics promote post-injury regeneration in ageing through metabolic modulation. Nat. Nanotechnol..

[bb0360] Liu P., Wang W., Li Z., Li Y., Yu X., Tu J., Zhang Z. (2022). Ferroptosis: a new regulatory mechanism in osteoporosis. Oxidative Med. Cell. Longev..

[bib761] Liao J., Fan L., Li Y., Xu Q.Q., Xiong L.Y., Zhang S.S., Liu J.H., Xiao Z.C., Zhang C., Yang J., Chen Z.S., Xiao K., Wang T.F., Lu Y. (2023). Recent advances in biomimetic nanodelivery systems: new brain-targeting strategies. J. Control. Release.

[bib773] Liao Jun, Gong Lidong, Xu Qingqiang, Wang Jingya, Yang Yuanyuan, Zhang Shiming, Dong Junwei (2024). Revolutionizing neurocare: biomimetic nanodelivery via cell membranes. Adv. Mater..

[bib762] Liao Jun, He Wenxiu, Zhang Shiming, Wang Jingya, Hansen Cheng, Gong Lidong, Li Yanglonghao (2025). Magnetic field driven ceria nanosystems for mitochondria targeted therapy of ischemic stroke. Adv. Funct. Mater..

[bib776] Liao J., Huang Q., Li R., Gong L., Li T., Lin Z. (2026). Long-acting nanomedicine for brain diseases. J. Control. Release.

[bib766] Liao Jun, Lin Zhiqiang, Chuang Liu (2026). Programming extracellular protein fate with peptide chimeras. Chem.

[bib775] Liao Jun, Lin Zhiqiang, Chuang Liu (2026). Precision uterine mRNA therapy to Restore implantation and fertility. Mater. Today.

[bib763] Liao J., Liu H., Li N. (2026). Ligand-triggered topology switching converts transient recognition into durable nanofibrillar anchoring. J. Am. Chem. Soc..

[bb0365] Liu J., Liu L., Li Y., Cai Z., Zhang H. (2023). Concordance of bone culture and deep tissue culture during the operation of diabetic foot osteomyelitis and clinical characteristics of patients. Eur. J. Trauma Emerg. Surg..

[bb0370] Maheshwari R., Kapoor D.U., Polaka S., Bhattacharya S., Prajapati B.G. (2025). Roadmap for commercial nanomedicine development: integrating quality by design principles with pharmaceutical nanotechnology. Mol. Pharm..

[bb0375] Matsuo K., Irie N. (2008). Osteoclast-osteoblast communication. Arch. Biochem. Biophys..

[bb0380] Ming Y., He X., Zhao Z., Meng X., Zhu Y., Tan H., Yang G., Hu Y., Zheng L. (2024). Nanocarrier-assisted delivery of berberine promotes diabetic alveolar bone regeneration by scavenging ROS and improving mitochondrial dysfunction. Int. J. Nanomedicine.

[bb0385] Miyazaki T. (2013). Design of bone-integrating organic-inorganic composite suitable for bone repair. Front. Biosci. (Elite Ed.).

[bb0390] Monti F., Perazza F., Leoni L., Stefanini B., Ferri S., Tovoli F., Zavatta G., Piscaglia F., Petroni M.L., Ravaioli F. (2024). RANK-RANKL-OPG axis in MASLD: current evidence linking bone and liver diseases and future perspectives. Int. J. Mol. Sci..

[bb0395] Moosavifar M., Parsaei H., Hosseini S., Mirmontazeri S.M., Ahadi R., Ahadian S., Engel F.B., Roshanbinfar K. (2022). Biomimetic organic-inorganic nanocomposite scaffolds to regenerate cranial bone defects in a rat animal model. ACS Biomater. Sci. Eng..

[bb0400] Moradi M., Salehi Barough M., Moghaddam-Banaem L., Johari Daha F., Rajabi-Moghadam S. (2025). Development of a potential bone-seeking radiopharmaceutical by sodium pyrophosphate labeled ^188^Rhenium (^188^Re-PYP) for bone pain palliation. Iran J. Pharm. Res..

[bb0405] Muñoz M., Robinson K., Shibli-Rahhal A. (2020). Bone health and osteoporosis prevention and treatment. Clin. Obstet. Gynecol..

[bb0410] Nadar R.A., Margiotta N., Iafisco M., van den Beucken J., Boerman O.C., Leeuwenburgh S.C.G. (2017). Bisphosphonate-functionalized imaging agents, anti-tumor agents and nanocarriers for treatment of bone cancer. Adv. Healthc. Mater..

[bb0415] Oh W.T., Yang Y.S., Xie J., Ma H., Kim J.M., Park K.H., Oh D.S., Park-Min K.H., Greenblatt M.B., Gao G., Shim J.H. (2023). WNT-modulating gene silencers as a gene therapy for osteoporosis, bone fracture, and critical-sized bone defects. Mol. Ther..

[bb0420] Ortega-Oller I., Padial-Molina M., Galindo-Moreno P., O’Valle F., Jódar-Reyes A.B., Peula-García J.M. (2015). Bone regeneration from PLGA micro-nanoparticles. Biomed. Res. Int..

[bb0425] Palacios S., Mejía A. (2015). Antiresorptives and anabolic therapy in sequence or combination for postmenopausal osteoporosis. Climacteric.

[bib770] Pan Y., Li L., Cao N., Liao J., Chen H., Zhang M. (2025). Advanced nano delivery system for stem cell therapy for Alzheimer’s disease. Biomaterials.

[bb0430] Parks N.J., Kawakami T.G., Avila M.J., White R., Cain G.R., Raaka S.D., Hornoff W., Fisher P., Moore P., Seibert J.A. (1993). Bone marrow transplantation in dogs after radio-ablation with a new Ho-166 amino phosphonic acid bone-seeking agent (DOTMP). Blood.

[bb0435] Peralta-Cuevas E., Degollado-Hernández N.Y., Martínez-Ortiz I.C., Gutierrez-Onofre A.J., Garcia-Atutxa I., Villanueva-Flores F. (2026). How do nanoparticle properties shape pharmacokinetics and pharmacodynamics? A mechanistic review. Front. Pharmacol..

[bb0440] Porter J.R., Ruckh T.T., Popat K.C. (2009). Bone tissue engineering: a review in bone biomimetics and drug delivery strategies. Biotechnol. Prog..

[bb0445] Qiao K., Xu L., Tang J., Wang Q., Lim K.S., Hooper G., Woodfield T.B.F., Liu G., Tian K., Zhang W., Cui X. (2022). The advances in nanomedicine for bone and cartilage repair. J. Nanobiotechnol..

[bb0450] Rachner T.D., Khosla S., Hofbauer L.C. (2011). Osteoporosis: now and the future. Lancet.

[bb0455] Rahmani N.R., Jahanmard F., Hassani Najafabadi A., Flapper J., Dogan O., Khodaei A., Storm G., Croes M., Kruyt M.C., Gawlitta D., Weinans H., Mastrobattista E., Amin Yavari S. (2025). Local delivery of lipid-based nanoparticles containing microbial nucleic acid for osteoimmunomodulation. Eur. J. Pharm. Sci..

[bb0460] Raisz L.G. (2005). Pathogenesis of osteoporosis: concepts, conflicts, and prospects. J. Clin. Invest..

[bb0465] Raje N.S., Bhatta S., Terpos E. (2019). Role of the RANK/RANKL pathway in multiple myeloma. Clin. Cancer Res..

[bb0470] Ramchand S.K., Leder B.Z. (2024). Sequential therapy for the long-term treatment of postmenopausal osteoporosis. J. Clin. Endocrinol. Metab..

[bb0475] Rong X., Kou Y., Zhang Y., Yang P., Tang R., Liu H., Li M. (2022). ED-71 prevents glucocorticoid-induced osteoporosis by regulating osteoblast differentiation via Notch and Wnt/β-catenin pathways. Drug Des. Dev. Ther..

[bb0480] Rosen C.J. (2003). The cellular and clinical parameters of anabolic therapy for osteoporosis. Crit. Rev. Eukaryot. Gene Expr..

[bb0485] Rossini M., Gatti D., Adami S. (2013). Involvement of WNT/β-catenin signaling in the treatment of osteoporosis. Calcif. Tissue Int..

[bb0490] Saker R., Regdon G., Sovány T. (2024). Pharmacokinetics and toxicity of inorganic nanoparticles and the physicochemical properties/factors affecting them. J. Drug Delivery Sci. Technol..

[bb0495] Samadi A., Salati M.A., Safari A., Jouyandeh M., Barani M., Singh Chauhan N.P., Golab E.G., Zarrintaj P., Kar S., Seidi F., Hejna A., Saeb M.R. (2022). Comparative review of piezoelectric biomaterials approach for bone tissue engineering. J. Biomater. Sci. Polym. Ed..

[bb0500] Seong S., Vijayan V., Kim J.H., Kim K., Kim I., Cherukula K., Park I.K., Kim N. (2023). Nano-formulations for bone-specific delivery of siRNA for CrkII silencing-induced regulation of bone formation and resorption to maximize therapeutic potential for bone-related diseases. Biomater. Sci..

[bb0505] Shadjou N., Hasanzadeh M. (2015). Bone tissue engineering using silica-based mesoporous nanobiomaterials: recent progress. Mater. Sci. Eng. C Mater. Biol. Appl..

[bb0510] Shen Y., Gwak H., Han B. (2024). Advanced manufacturing of nanoparticle formulations of drugs and biologics using microfluidics. Analyst.

[bib765] Shi X., Zhao Y., Gao H.Y., Yang W., Liao J., Wang H.H., Wang X.T., Yan W. (2025). NFS1, together with FXN, protects cells from ferroptosis and DNA damage in diffuse large B-cell lymphoma. Redox Biol..

[bb0515] Sims N.A., Martin T.J. (2020). Osteoclasts provide coupling signals to osteoblast lineage cells through multiple mechanisms. Annu. Rev. Physiol..

[bb0520] Sisay M., Mengistu G., Edessa D. (2017). The RANK/RANKL/OPG system in tumorigenesis and metastasis of cancer stem cell: potential targets for anticancer therapy. Onco. Targets Ther..

[bb0525] Sølling A.S., Langdahl B.L., Cosman F. (2026). Recent advances in osteoporosis therapeutics. Annu. Rev. Med..

[bb0530] Souto E.B., Blanco-Llamero C., Krambeck K., Kiran N.S., Yashaswini C., Postwala H., Severino P., Priefer R., Prajapati B.G., Maheshwari R. (2024). Regulatory insights into nanomedicine and gene vaccine innovation: Safety assessment, challenges, and regulatory perspectives. Acta Biomater..

[bb0535] Subarajan P., Arceo-Mendoza R.M., Camacho P.M. (2024). Postmenopausal osteoporosis: a review of latest guidelines. Endocrinol. Metab. Clin. N. Am..

[bb0540] Sun J., Zhao D., Wang Y., Chen P., Xu C., Lei H., Wo K., Zhang J., Wang J., Yang C., Su B., Jin Z., Luo Z., Chen L. (2023). Temporal immunomodulation via wireless programmed electric cues achieves optimized diabetic bone regeneration. ACS Nano.

[bb0545] Takeda Y.S., Wang M., Deng P., Xu Q. (2016). Synthetic bioreducible lipid-based nanoparticles for miRNA delivery to mesenchymal stem cells to induce neuronal differentiation. Bioeng. Transl. Med..

[bb0550] Tao H., Li W., Zhang W., Yang C., Zhang C., Liang X., Yin J., Bai J., Ge G., Zhang H., Yang X., Li H., Xu Y., Hao Y., Liu Y., Geng D. (2021). Urolithin a suppresses RANKL-induced osteoclastogenesis and postmenopausal osteoporosis by, suppresses inflammation and downstream NF-κB activated pyroptosis pathways. Pharmacol. Res..

[bb0555] Tateiwa D., Nakagawa S., Tsukazaki H., Okada R., Kodama J., Kushioka J., Bal Z., Ukon Y., Hirai H., Kaito T. (2021). A novel BMP-2-loaded hydroxyapatite/beta-tricalcium phosphate microsphere/hydrogel composite for bone regeneration. Sci. Rep..

[bb0560] Tella S.H., Gallagher J.C. (2014). Prevention and treatment of postmenopausal osteoporosis. J. Steroid Biochem. Mol. Biol..

[bb0565] Thangavel M., Elsen Selvam R. (2022). Review of physical, mechanical, and biological characteristics of 3D-printed bioceramic scaffolds for bone tissue engineering applications. ACS Biomater. Sci. Eng..

[bb0570] Tylawsky D.E., Kiguchi H., Vaynshteyn J., Gerwin J., Shah J., Islam T., Boyer J.A., Boué D.R., Snuderl M., Greenblatt M.B., Shamay Y., Raju G.P., Heller D.A. (2023). P-selectin-targeted nanocarriers induce active crossing of the blood-brain barrier via caveolin-1-dependent transcytosis. Nat. Mater..

[bb0575] U.S. Food and Drug Administration (2018).

[bb0580] van Dam P.A., Verhoeven Y., Trinh X.B. (2020). The non-bone-related role of RANK/RANKL signaling in cancer. Adv. Exp. Med. Biol..

[bb0585] VandenBerg M.A., Dong X., Smith W.C., Tian G., Stephens O., O’Connor T.F., Xu X. (2025). Learning from the future: towards continuous manufacturing of nanomaterials. AAPS Open.

[bb0590] Vishal M., Ajeetha R., Keerthana R., Selvamurugan N. (2016). Regulation of Runx2 by histone deacetylases in bone. Curr. Protein Pept. Sci..

[bb0595] Wang L.T., Chen L.R., Chen K.H. (2023). Hormone-related and drug-induced osteoporosis: a cellular and molecular overview. Int. J. Mol. Sci..

[bb0600] Wang S., Shi X., Xiong T., Chen Q., Yang Y., Chen W., Zhang K., Nan Y., Huang Q., Ai K. (2024). Inhibiting mitochondrial damage for efficient treatment of cerebral ischemia-reperfusion injury through sequential targeting nanomedicine of neuronal mitochondria in affected brain tissue. Adv. Mater..

[bb0605] Wang X., Qu Z., Zhao S., Luo L., Yan L. (2024). Wnt/β-catenin signaling pathway: proteins’ roles in osteoporosis and cancer diseases and the regulatory effects of natural compounds on osteoporosis. Mol. Med..

[bb0610] Wang C., Zhang Y., Kong W., Rong X., Zhong Z., Jiang L., Chen S., Li C., Zhang F., Jiang J. (2024). Delivery of miRNAs using nanoparticles for the treatment of osteosarcoma. Int. J. Nanomedicine.

[bib772] Wang L., Gu M., Zhang X., Kong T., Liao J., Zhang D., Li J. (2025). Recent advances in nanoenzymes based therapies for glioblastoma: overcoming barriers and enhancing targeted treatment. Adv. Sci..

[bb0615] Wang W., Ma A., Zhai C., Lan W., Liu Z., Yang Z., Zhang Y., Zhu T., Yu T., Lan J. (2025). Bisphenol A and Di-n-butyl phthalate disrupt bone homeostasis bidirectionally via CD36-mediated BMSCs autophagy inhibition and exosome-promoted osteoclastogenesis. J. Hazard. Mater..

[bb0620] Wang Q., Feng K., Wan G., Liao W., Jin J., Wang P., Sun X., Wang W., Jiang Q. (2025). A ROS-responsive hydrogel encapsulated with matrix metalloproteinase-13 siRNA nanocarriers to attenuate osteoarthritis progression. J. Nanobiotechnol..

[bb0625] Wen W., Guo P., Xue H.Y., Lun Wong H. (2022). Development of local injectable, bone-targeting nanocarriers of triptolide for treatment of bone-only metastasis. Int. J. Pharm..

[bb0630] Wu D., Cline-Smith A., Shashkova E., Perla A., Katyal A., Aurora R. (2021). T-cell mediated inflammation in postmenopausal osteoporosis. Front. Immunol..

[bib777] Wu Zhaoqi, Zhenle Su, Huang Jun, Martin Gluchman, Chenyu Zhao (2025). The burden and trends of soft tissue and extraosseous sarcomas in China: an observational study from 1990 to 2021. Med. Adv..

[bb0635] Wu Z., Zhu J., Wen Y., Lei P., Xie J., Shi H., Wu R., Lou X., Hu Y. (2023). Hmga1-overexpressing lentivirus protects against osteoporosis by activating the Wnt/β-catenin pathway in the osteogenic differentiation of BMSCs. FASEB J..

[bib778] Wu Yimao, Sun Ruowei, Ren Shuai, Gokhan Zengin, Mengyao Li (2025). Neuronal reshaping of the tumor microenvironment in tumorigenesis and metastasis: bench to clinic. Med. Adv..

[bb0640] Wu Y., Sun B., Tang Y., Shen A., Lin Y., Zhao X., Li J., Monteiro M.J., Gu W. (2024). Bone targeted nano-drug and nano-delivery. Bone Res..

[bb0645] Wu X., Jin S., Wang Q., Chen L., Cai X., Yu M., Guo H., Zhang H., Liu H., Li C., Zhang S., Shi X., Feng L., Gong S., Luo D., Wang C., Liu Y. (2026). Calcium phosphate nanoparticle-immobilized macrophage-derived extracellular vesicle nanohybrid facilitates diabetic bone regeneration. Adv. Mater..

[bb0650] Xiang T., Zhou Z., Li Y., Suo Y., Dai J., Shi X., Zhou X., Sheng L. (2025). Loss of SMN impairs osteoblast-osteoclast coupling via IGF1-Akt-OPG axis in spinal muscular atrophy. FASEB J..

[bb0655] Xie X., Yang J., Ding C., Li Y., Ai-Smadi F., Zha K., Lin C., Lin Z., Yu C., Zeng R., Hu W., Liao J., Ouyang L., Xia T., Zhao P., Mi B., Liu G. (2025). Holistic regulation of the diabetic osteo-microenvironment via NIR-II nanocarriers with dual NO/pH responsiveness for enhanced bone regeneration. Bioact. Mater..

[bb0660] Xiong Y., Mi B.B., Shahbazi M.A., Xia T., Xiao J. (2024). Microenvironment-responsive nanomedicines: a promising direction for tissue regeneration. Mil. Med. Res..

[bb0665] Xu H., Lu X., Li M., Huang X., Yao N., Gan H., Huang X., Zhao Z., Hu Z., Zhao X., Lai Y., Li M., Chen S., Chen Y., Huang D. (2024). Jiangu formula: a novel osteoclast-osteoblast coupling agent for effective osteoporosis treatment. Phytomedicine.

[bib774] Yang Shiyu, Chen Heming, Su Wei, Luo Yunchun, Liao Jun, Wang Yun, Xiong Liyan (2023). Protective effects of Salvianic acid A against multiple-organ ischemia-reperfusion injury: a review. Front. Pharmacol..

[bb0670] Yang L., Wang K., Zeng Z.H., Zhao H.E., Bai L.M. (2025). Morroniside improves diabetic osteoporosis via the AGE/RAGE/Wnt/β-catenin signaling pathway. Kaohsiung J. Med. Sci..

[bb0675] Yao Y., Cai X., Chen Y., Zhang M., Zheng C. (2025). Estrogen deficiency-mediated osteoimmunity in postmenopausal osteoporosis. Med. Res. Rev..

[bb0680] Ye G., Bao F., Zhang X., Song Z., Liao Y., Fei Y., Bunpetch V., Heng B.C., Shen W., Liu H., Zhou J., Ouyang H. (2020). Nanomaterial-based scaffolds for bone tissue engineering and regeneration. Nanomedicine (London).

[bb0685] You Y., Leng S., Shi J., Yang H., Chang M., Ma Q., Zhang D., Sun H., Wang L., Gao Z., Cui J., Liu X. (2025). Integration of bone-targeted delivery and crosstalk modulation of liver-bone axis for improved osteoporosis therapy. ACS Nano.

[bb0690] Yu B., Wang C.Y. (2022). Osteoporosis and periodontal diseases - an update on their association and mechanistic links. Periodontology.

[bb0695] Yu J., Dan N., Eslami S.M., Lu X. (2024). State of the art of silica nanoparticles: an overview on biodistribution and preclinical toxicity studies. AAPS J..

[bb0700] Zhang F., Cho W.C. (2023). Therapeutic potential of RUNX1 and RUNX2 in bone metastasis of breast cancer. Expert Opin. Ther. Targets.

[bb0705] Zhang W., Gao R., Rong X., Zhu S., Cui Y., Liu H., Li M. (2022). Immunoporosis: role of immune system in the pathophysiology of different types of osteoporosis. Front. Endocrinol. (Lausanne).

[bb0710] Zhang Y., Zhu Y., Habibovic P., Wang H. (2024). Advanced synthetic scaffolds based on 1D inorganic micro-/nanomaterials for bone regeneration. Adv. Healthc. Mater..

[bb0715] Zhang W., Zhang Y., Hao Z., Yao P., Bai J., Chen H., Wu X., Zhong Y., Xue D. (2025). Synthetic nanoparticles functionalized with cell membrane-mimicking, bone-targeting, and ROS-controlled release agents for osteoporosis treatment. J. Control. Release.

[bib779] Yuan Han. (2025). Agentic large language models for healthcare: current progress and future opportunities. Med. Adv..

[bib764] Zang J., Ren Z., Wang H., Yang X., Song Y., Liao J., Li X. (2025). Emerging nanozyme strategies for precision breast cancer treatment. Adv. Sci..

[bb0720] Zhang X., Chan H.W., Shao Z., Wang Q., Chow S., Chow S.F. (2025). Navigating translational research in nanomedicine: a strategic guide to formulation and manufacturing. Int. J. Pharm..

[bb0725] Zhong J., Wen W., Wang J., Zhang M., Jia Y., Ma X., Su Y.X., Wang Y., Lan X. (2023). Bone-targeted dual functional lipid-coated drug delivery system for osteosarcoma therapy. Pharm. Res..

[bb0730] Zhou M., Graves D.T. (2022). Impact of the host response and osteoblast lineage cells on periodontal disease. Front. Immunol..

[bb0735] Zhou S., Xiao C., Fan L., Yang J., Ge R., Cai M., Yuan K., Li C., Crawford R.W., Xiao Y., Yu P., Deng C., Ning C., Zhou L., Wang Y. (2024). Injectable ultrasound-powered bone-adhesive nanocomposite hydrogel for electrically accelerated irregular bone defect healing. J. Nanobiotechnol..

[bb0740] Zhu X., Li S. (2023). Nanomaterials in tumor immunotherapy: new strategies and challenges. Mol. Cancer.

[bib769] Zhu Y., Xi Q., Liu Y., Zhou Y., Liao J., Wu Q. (2026). Recent advances in exosome-based nanodelivery systems for Parkinson’s disease. Biomaterials.

[bb0745] Ziros P.G., Basdra E.K., Papavassiliou A.G. (2008). Runx2: of bone and stretch. Int. J. Biochem. Cell Biol..

[bb0750] Zorrón M., Cabrera A.L., Sharma R., Radhakrishnan J., Abbaszadeh S., Shahbazi M.A., Tafreshi O.A., Karamikamkar S., Maleki H. (2024). Emerging 2D nanomaterials-integrated hydrogels: advancements in designing theragenerative materials for bone regeneration and disease therapy. Adv. Sci. (Weinh.).

[bb0755] Zou T., Jaladanki S.K., Liu L., Xiao L., Chung H.K., Wang J.Y., Xu Y., Gorospe M., Wang J.Y. (2016). H19 long noncoding RNA regulates intestinal epithelial barrier function via microRNA 675 by interacting with RNA-binding protein HuR. Mol. Cell. Biol..

[bb0760] Zou J., Yang W., Cui W., Li C., Ma C., Ji X., Hong J., Qu Z., Chen J., Liu A., Wu H. (2023). Therapeutic potential and mechanisms of mesenchymal stem cell-derived exosomes as bioactive materials in tendon-bone healing. J. Nanobiotechnol..

